# Belowground Communities in Lowlands Are Less Stable to Heat Extremes Across Seasons

**DOI:** 10.1111/ele.70225

**Published:** 2025-10-03

**Authors:** Gerard Martínez‐De León, Ludovico Formenti, Jörg‐Alfred Salamon, Madhav P. Thakur

**Affiliations:** ^1^ Institute of Ecology and Evolution University of Bern Bern Switzerland; ^2^ Institute of Animal Ecology & Field Station Schapen University of Veterinary Medicine Hannover Hannover Germany

**Keywords:** Collembola, connectance, elevation, fungi, joint species distribution model, phenology, range contraction, recovery, resistance, thermal vulnerability

## Abstract

Ecological responses to climate extremes vary drastically in different spatiotemporal contexts. Here, we investigate how soil communities at high‐ and low‐elevation sites respond to extreme heat events in different seasons (spring, summer and autumn). We simulated 1‐week heat events based on site‐specific climatic history in laboratory experiments using 360 field‐collected soil cores and measured the resistance and recovery of two major groups of soil biota: Collembola and fungi. We found that Collembola communities from low elevations exhibited the lowest resistance to extreme heat in spring and summer, with full recovery occurring primarily in spring soils. Fungal communities remained generally stable, though pathogens increased their relative abundances following summer heat events. Network analysis revealed increased connectance of negative associations between Collembola and fungi in response to extreme heat. We provide experimental evidence for how heat events can restructure and destabilise ecological communities depending on spatiotemporal contexts like elevation and seasonality.

## Graphical abstract

We experimentally exposed soil communities collected at different elevations and seasons to extreme heat events –based on site‐specific climatic history‐ and measured the stability (resistance and recovery) of Collembola and fungi, representing key trophic groups in belowground food chains. Our results show that lowland communities responded strongly to the extreme heat events, while highland communities remained largely unaltered. Remarkably, lowland communities recovered better in spring than in summer, underscoring the importance of the seasonal context in determining ecological stability to climate extremes.

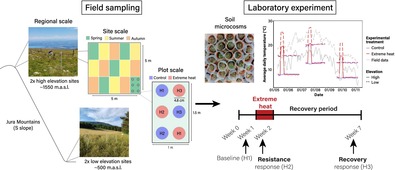

## 1 Introduction

Climate change is increasing extreme events with significant ecological impacts (Harris et al. 2018; IPCC 2023; Thakur et al. 2022). Such extremes, particularly heat events, can push organisms beyond their adaptive capacities by exceeding physiological thermal optima and reducing performance (Ma et al. 2020; Williams et al. 2016). The degree of short‐term vulnerability to extreme heat (resistance) is determined by both the magnitude of thermal change experienced (exposure) and the resulting fitness response (sensitivity) (Buckley and Kingsolver 2021; Martínez‐De León and Thakur 2024; Williams et al. 2008). Thermal vulnerability varies latitudinally, with tropical and mid‐latitude ectotherms being more susceptible despite having similar heat tolerances to higher‐latitude organisms (Sunday et al. 2019), as they live closer to their thermal limits (Deutsch et al. 2008; Kingsolver et al. 2013). When scaling from organismal to population and community levels, additional factors influence thermal vulnerability (Louthan et al. 2021), including the seasonal timing of heat events (Cinto Mejía and Wetzel 2023; Jentsch et al. 2007).

The ecological significance of the timing of extreme events depends on the exposure of heat‐sensitive life‐history processes (e.g., juvenile survival (Ma et al. 2018), reproduction (Walsh et al. 2019)). Specifically, the impact of extreme heat will be amplified when it coincides with key phenological periods (Cinto Mejía and Wetzel 2023; Forrest and Miller‐Rushing 2010), affecting long‐term ecological dynamics such as population recovery (Martínez‐De León et al. 2024; Martínez‐De León and Thakur 2024). For example, when heat extremes occur during reproductive periods, recruitment may be able to compensate for heat‐induced impacts on adult survival (Coblentz et al. 2024). However, such impacts may persist in the long term if demographic buffering capacity is exceeded (Hilde et al. 2020) and compensation is disrupted (Coblentz et al. 2024), especially when survival is impacted and additional breeding attempts are no longer feasible (e.g., late in the reproductive period) (Isotalo et al. 2022). Key phenological periods are not only seasonally dependent but also change spatially, as they are shaped by local climatic conditions (Roslin et al. 2021). Thus, given that phenology and thermal vulnerability vary across geographic gradients (Louthan et al. 2021; Roslin et al. 2021), the ecological consequences of extreme heat events could differ depending on both the seasonal timing and the geographical context. Yet, these important spatial and temporal ecological dimensions have rarely been considered in comparative studies of thermal vulnerability, despite their potential to interactively influence short‐ and long‐term ecological stability to extreme heat events.

Elevational gradients provide unique opportunities to examine variation in ecological responses to extreme heat events (Sundqvist et al. 2013). Local climatic conditions vary radically over short distances across elevations as a result of temperature lapse rates (Körner 2007), and, in many temperate environments, due to orographic precipitation (Hodkinson 2005). These abiotic factors are drivers of phenology at the site scale (Forrest and Miller‐Rushing 2010), and thereby generate variation in phenological patterns across elevations (Hodkinson 2005). For instance, in temperate ecosystems, high‐elevation organisms have short activity periods condensed around the summer months (Forrest and Miller‐Rushing 2010; Hodkinson 2005). In turn, low‐elevation organisms have longer activity periods, only interrupted in dry summers and in the winter months. These distinct phenological patterns may underlie dissimilar periods of high thermal vulnerability and, therefore, the impact of seasonal timing of extreme heat events is expected to depend on the elevation. For example, at low elevations, hot conditions during summer can significantly alter organismal survival (Buckley et al. 2021). However, avoidance strategies commonly displayed by low‐elevation organisms, such as seasonal escape or induced diapause, may enable them to evade the effects of extreme heat (Kefford et al. 2022; Sgrò et al. 2016). At higher elevations, summer is typically a favourable period for reproduction and recruitment, but these processes could be compromised if temperatures during extreme heat events exceed reproductive thermal limits (Walsh et al. 2019).

Within a given community, there is enormous variation across different taxa in their life histories and thermal responsiveness (Berg et al. 2010; Franken et al. 2018), potentially leading to trophic mismatches after extreme heat events (Thackeray et al. 2010; Thakur 2020). In belowground communities, fungi are key drivers of ecosystem functioning (Delgado‐Baquerizo, Reich, et al. 2020) and represent important resources for invertebrate consumers, especially for microbivores like Collembola (Pollierer and Scheu 2021; Potapov et al. 2016). Fungi form the foundation of the slow energy channel in soil food webs (Moore and Hunt 1988; Thakur and Geisen 2019). Consequently, fungal communities are relatively resistant to climate extremes (e.g., heat and drought (Knight et al. 2024; de Vries et al. 2018)), although they tend to recover slowly (de Vries et al. 2012). Given the overall stability of fungal communities to climate extremes, they can represent readily available resources for recovering populations of invertebrate consumers like Collembola, thereby promoting overall food web stability (Bardgett and Caruso 2020). However, the increasing severity of climate extremes could affect fungal responses in the long term (Cordero et al. 2023; Knight et al. 2024), constraining the recovery of consumers. Besides, climate‐driven shifts in fungal communities could result in increased dominance of species that represent poor‐quality resources (because of e.g., low palatability or nutritional value) (Sanders et al. 2024) or even pathogens (Delgado‐Baquerizo, Guerra, et al. 2020), further limiting the recovery of soil Collembola. The structure of association networks between Collembola and fungi can therefore yield insights into their responses to extreme events. Specifically, more prevalent positive associations between Collembola and fungi after extreme heat (i.e., more connectance, indicating more generalised associations) (Petchey et al. 2010) can be expected, as Collembola might become more reliant on fungal resources to sustain their populations. Correspondingly, negative Collembola‐fungal associations could also become more frequent after extreme heat, as a result of climate‐driven increases of fungi representing low‐quality resources and/or pathogenic species (Sanders et al. 2024), thus limiting the recovery of Collembola species.

**FIGURE 1:**
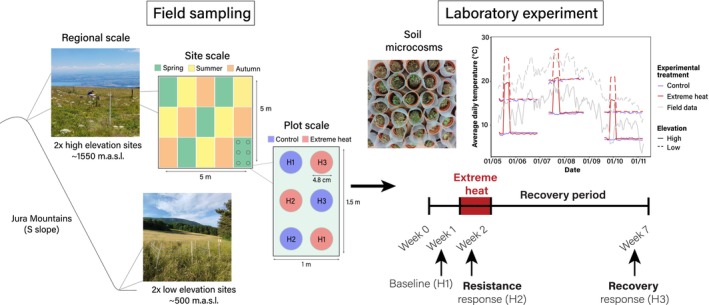
Scheme of the experimental design of the study. We used a split‐plot sampling design (left side of the figure), whereby samples (intact soil cores) were taken from two regional‐scale blocks, each containing one high‐ and one low‐elevation site (Figure S1). Sites were defined as a delineated 5 × 5 m area representative of the dry grasslands of the study region (pictures in Figure S2). Within sites and seasons (i.e., spring, summer, autumn), six soil cores were obtained from each of five 1 × 1.5 m plots. The sampling locations of data‐level predictors (temperature regimes and harvests) were randomised within each plot, whereas the sampling locations of plot‐level predictors (seasons) were kept constant in all sites to avoid the sampling from adjacent plots in the same season. The pictures displayed in the figure were taken in the summer season from one of our high (above: Chasseron) and low (below: Onnens) elevation sites (site‐specific information is provided in Table S1). The colours of the plots (site scale) denote different sampling seasons: Spring (green), summer (yellow) and autumn (orange). The circles shown at the plot scale represent the soil cores used as microcosms in the laboratory experiment (right side of the figure), which were allocated to one of two temperature treatments (control: Blue; extreme heat: Red) and one of three harvests (H1: Baseline or harvest 1; H2: Resistance phase or harvest 2; H3: Recovery phase or harvest 3). All harvests were destructive, meaning experimental replications were true for each harvest. The size of the soil cores relative to the plot is enhanced for visualisation purposes. Average daily soil temperatures (depth 3–5 cm) measured over the course of the laboratory experiments are shown, together with the temperatures recorded in the field sites during the same period (6 May to 9 November 2022). Mean temperatures from the two sites at the same elevation are displayed as grey lines; site‐specific temperature values are provided in Figure S3.

Here, we investigated how belowground communities respond to extreme heat events, using intact soil cores collected from temperate grasslands at two different elevations (spanning ~1000 m of altitude difference) and across three seasons (spring, summer, autumn) (Figure 1). We exposed these field‐collected soil cores to 1‐week extreme heat events in controlled laboratory conditions, and tracked the responses of two trophic levels (Collembola and fungi) at the end of extreme heat (i.e., resistance response) and after a 5‐week recovery period (i.e., recovery response) ‐representing the generation time of several Collembola species. We examined how the extreme heat events altered total abundances, species‐specific abundances (using joint species distribution models), diversity indices and bipartite association networks of Collembola and fungi. Our hypotheses are (1) that heat events reaching higher temperatures (e.g., low elevation sites in summer) will induce more negative responses, given that the thermal safety margins of organisms are narrower (i.e., closer to their thermal limits) and metabolic costs are greater at high absolute temperatures (Deutsch et al. 2008; Dillon et al. 2010). Moreover, we expect that (2) both negative resistance and recovery responses to extreme heat events are more likely in cold‐adapted organisms (e.g., typical high‐elevation species; Martínez‐De León et al. 2024), and in species permanently living belowground (Thakur et al. 2023). We finally anticipate (3) heat‐induced increases in the connectance of Collembola‐fungi association networks, either for positive associations (indicating increased reliance of Collembola on a broader range of fungal resources; Petchey et al. 2010) and/or negative ones (indicative of greater limitation of Collembola by low‐quality resources or pathogens; Sanders et al. 2024).

## 2 Materials and Methods

### 2.1 Field Sites and Experimental Design

The study area was located in the Swiss Jura Mountains, consisting of two blocks located ca. 40 km apart (Figure S1). Each block had two sites at contrasting elevations: low (ca. 500 m.a.s.l.) and high elevation (ca. 1550 m.a.s.l.) (Figure 1; Figures S1 and S2). All sites were located in extensively managed dry meadows representative of the study area, on south‐facing slopes and with no recent soil disturbances (Tables S1 and S2). Soil temperatures (5 cm depth) were monitored at 30‐min intervals throughout the duration of the study (6 May to 9 November 2022) using data loggers (HOBO Pendant MX, Onset Computer Corporation, USA) (Figure S3).

Our experimental units were intact soil cores (diameter 4.8 cm, depth 5.5 cm; Vienna Scientific Instruments, Austria) obtained in 2022 at three different seasons: spring (6–9 May), summer (4–7 July) and autumn (13–16 September). We used a split‐plot experimental design (Quinn and Keough 2002), composed of three grouping factors (block, site and plot), as well as predictors at the site level (elevation), at the plot level (season) and at the sample level (temperature regime and harvest (Schielzeth and Nakagawa 2013)) (Figure 1). Within each site and season, we sampled five plots of 1.5 × 1 m. We collected six soil cores from each plot and randomly allocated them to the experimental treatments: one of the two temperature treatments (control conditions vs. extreme heat; details in Temperature treatments) and one of the three destructive harvests (details in Data collection). We therefore established a total of 360 experimental units: 2 elevations × 2 sites (nested within elevation) × 3 seasons × 5 plots (nested within season) × 2 temperature treatments × 3 harvests. With this sampling design, we aimed to capture large‐scale variation in the composition of soil communities from different sites, hence enhancing the generality of our study while minimising small‐scale variation by sampling all experimental treatment combinations within the same plot (Figure 1).

Immediately after collecting the soil cores, we stored them in polypropylene pots (hereafter referred to as microcosms; Supplementary Methods S1), which were transported to the laboratory on the same day of field sampling, weighed and allocated to lit incubators set at their respective temperature regimes (details in the next section; Table S3). We maintained the same water content as at the time of sampling (Figure S4) during the entire duration of the experiment (except in the extreme heat treatment during the week of the heat event; details in the following section), by weighing each microcosm every third day and adjusting evaporative losses with deionised water.

### 2.2 Temperature Treatments

Ambient (control) temperatures in the incubators were set to simulate the average climatic conditions in the field sites, and were therefore adjusted to the corresponding elevation and season of the samples. We retrieved climatic data of the reference period 2015–2020 from two representative weather stations (one for each elevation, Table S3). This time reference was chosen due to the increasing frequency of heat waves in the region, especially in recent years (CH2018 2018). Ambient conditions were defined as the mean average daily temperatures of the 2 months that our microcosms were incubated in the laboratory. For example, samples collected in spring were exposed to the average temperature conditions of May and June as the ambient temperature in our lab experiment for the entire experimental duration of this season. To simulate heat events that were statistically extreme in all elevations and seasons (CH2018 2018; IPCC 2023), we calculated the 99th percentile of average daily temperature across the reference period (Jentsch et al. 2007), and applied this temperature during seven consecutive days (Figure 1). All ambient and extreme heat temperature values for each season and site are provided in Table S3. We additionally assessed how our experimental extreme heat events compared to naturally occurring heat extremes in the field sites during the study period (details in Table S4).

To imitate typically dry conditions encountered during extreme heat events, microcosms allocated to the extreme heat treatment did not receive any water inputs during the week of the heat event, and water losses were compensated only at the start of the recovery phase. All temperature regimes adopted a diel light and temperature cycle (8 h night/16 h day), with a 6°C‐amplitude between night and day (Table S3). Soil temperatures (depth 3–5 cm; Figure 1) were monitored in the incubators (SANYO MIR‐253, Japan) at 30‐min intervals (HOBO MX Multi‐Channel, Onset Computer Corporation, USA).

### 2.3 Data Collection

After field sampling, all soil microcosms were acclimated for 1 week in the incubators at ambient temperatures. We collected data on soil‐living communities of microarthropods (Collembola) and fungi across three harvests for each season: harvest 1 (before the extreme heat event), harvest 2 (immediately after the extreme heat event) and harvest 3 (after a 5‐week recovery period following the extreme heat event). At each harvest, we collected a scoop of moist soil from the bottom of each microcosm to minimise sample disturbance, rather than using the common practice of homogenising the sample (mean weight subsamples (g) ± SD: 8.55 ± 0.44). The subsamples were then stored at −20°C until extraction of fungal DNA (March to May 2023). Next, we extracted all microarthropods from the microcosms with gradual heating from 25°C up to 55°C for 7 days following the Macfayden extraction method (Macfadyen 1961). All animals were collected in glycol water solution (1:1) and later transferred to 70% ethanol.

Collembolans were identified to species level (Table S5) and assigned to one of three categories depending on their adaptations to occupy different depths of the soil profile: epedaphic (surface‐living), hemiedaphic (living in litter and upper soil layers) and euedaphic (permanently living in the soil).

### 2.4 Fungal ITS Metabarcoding

Fungal DNA was extracted from 250 mg of bulk fresh soil (subsamples) using the Qiagen DNAeasy PowerSoil Pro Kit, following the manufacturer's instructions. After PCR amplification, we sequenced the full ITS region (ITS1‐ITS2) with the PacBio Sequel II instrument (Pacific Biosciences, USA) at the Next Generation Sequencing Platform of the University of Bern. Processing of the HiFi reads was performed with the pb‐16S‐nf pipeline (https://github.com/PacificBiosciences/HiFi‐16S‐workflow), which makes use of QIIME2 (Bolyen et al. 2019) and DADA2 (Callahan et al. 2016). We then obtained the main trophic strategy of each fungal genus (i.e., saprotroph, symbiotroph, pathogenic) from the FungalTraits database (Põlme et al. 2020) (Supplementary Methods S2).

### 2.5 Data Analyses: Total Abundances and Diversity Indices

All analyses were performed in R version 4.4.0 (R Core Team 2024). We tested how the effects of extreme heat on belowground communities were modulated by elevation and season, using the following three‐way interaction model:

(1) $$\begin{array}{c} \text{Response variable}\sim \text{Elevation}\times \text{Season}\\ \;\times \text{Temperature treatment}+\left(1\;\right|\;\text{Site}) \end{array}$$

where Site (*N* = 4) was treated as a random factor in all models to control for non‐independence among experimental units at each site (Schielzeth and Nakagawa 2013). All models were fitted separately for each experimental harvest: harvest 1 or baseline, harvest 2 or resistance response and harvest 3 or recovery response (Figure 1). Linearity assumptions (i.e., normality of residuals, overdispersion, zero‐inflation, homogeneity of variance) were verified with the package DHARMa (Hartig 2022). We obtained marginal means and contrasts between control and extreme heat treatments using the emmeans package (Lenth 2024).

Total Collembola abundances were analysed with generalised linear mixed‐effects models (GLMM) with negative binomial distribution (Equation 1), using the R package glmmTMB (Brooks et al. 2017). We also employed negative binomial GLMMs to analyse the total number of reads for different groups of fungi according to their trophic strategy (saprotrophs, pathogens, symbionts and unassigned fungi), including the log‐transformed number of reads as a covariate to control for variation in sequencing depth across samples (Leite and Kuramae 2020; Tedersoo et al. 2022). Hence, given the compositional nature of sequencing data, the numbers of fungal reads represent relative abundances.

The diversity of Collembola and fungi was assessed using diversity profiles at three Hill numbers (order *q*): *q* = 0 (species richness), *q* = 1 (Shannon‐Hill) and *q* = 2 (Simpson‐Hill). We computed diversity estimates using coverage‐based rarefaction and extrapolation to equalise samples with the iNEXT package (Chao et al. 2014; Hsieh et al. 2022). The resulting point estimates of diversity were tested using linear mixed models (Equation 1) with a Gaussian distribution.

### 2.6 Data Analyses: Species Abundances and Association Networks

Species abundances were evaluated using joint species distribution models (jSDMs) (Ovaskainen et al. 2017; Warton et al. 2015) within the Hierarchical Modelling of Species Communities framework (package Hmsc; Tikhonov et al. 2020), assuming default prior distributions (Ovaskainen and Abrego 2020). The ecological interpretation of the parameters estimated with the jSDMs is shown in Table S6. Block (*N* = 2) was added as a random effect in all fitted jSDMs to account for variation in species occurrences driven by their large‐scale geographic distributions (see Figure S5). We adopted a prevalence threshold of 25% to discard rare taxa (i.e., species occurring in < 30 out of the 120 experimental units sampled at each harvest), which may provide low statistical power due to the scarcity of data (e.g., Burg et al. (2024)). We performed variance partitioning to extract the proportion of total variance explained by the experimental treatment (extreme heat), the natural variables (elevation and season) and the random effects (site and block). Three sets of models with different groups of response variables were delineated: (1) the Collembola model, measuring responses of Collembola communities; (2) the fungi model, assessing responses of fungal communities; and (3) the Collembola‐fungi models, examining associations between Collembola and fungi. First, in the Collembola model, we used the log‐normal Poisson distribution (analogous to negative binomial distribution) (Ovaskainen and Abrego 2020). We further modelled the influence of the species' traits on their abundance responses by including the species' vertical stratification (epedaphic, hemiedaphic and euedaphic). Second, in the fungal model, we accounted for zero‐inflation, as typically encountered in sequencing data, by constructing a hurdle model that consisted of two parts: presence‐absence (probit regression) and abundance conditional on presence (linear regression with normal distribution, using log‐transformed and scaled counts). We further controlled for variation in sequencing depth by including the log‐transformed number of reads as a covariate (Leite and Kuramae 2020; Tedersoo et al. 2022). We additionally included the fungal species' trophic strategy in the models (saprotrophs, symbionts, pathogens and unassigned) to examine how this trait can mediate fungal occurrence and relative abundance responses. MCMC convergence for all estimated parameters was assessed in terms of potential scale reduction factors (Table S7) (Gelman and Rubin 1992). All jSDMs were fitted with four chains of 250 samples each, yielding 1000 posterior samples in total.

The third set of jSDMs (Collembola‐fungi models) allowed us to estimate associations between Collembola and fungi, followed by the analysis of network properties (i.e., connectance) to summarise these associations at the network level. In short, we created separate subsets from the full dataset for each elevation and season, resulting in six subsets, each containing 20 samples. Given the very low prevalence of Collembola species in summer at low elevation, we could not determine associations in this case. Next, we built the jSDMs using fungal species relative abundances as response variables (log‐transformed and scaled abundances, conditional on presence), while treating Collembola species abundances (log‐transformed +1 and scaled) and their interactive effects with extreme heat as explanatory variables. We retained the associations between Collembola and fungi with 95% credible intervals not overlapping zero for control and extreme heat treatments, using the ci function from the bayestestR package (Makowski et al. 2019). Network connectance was finally calculated for positive and negative associations separately (Supplementary Methods S3).

## 3 Results

### 3.1 Collembola Communities: Total Abundance and Diversity Responses

**FIGURE 2:**
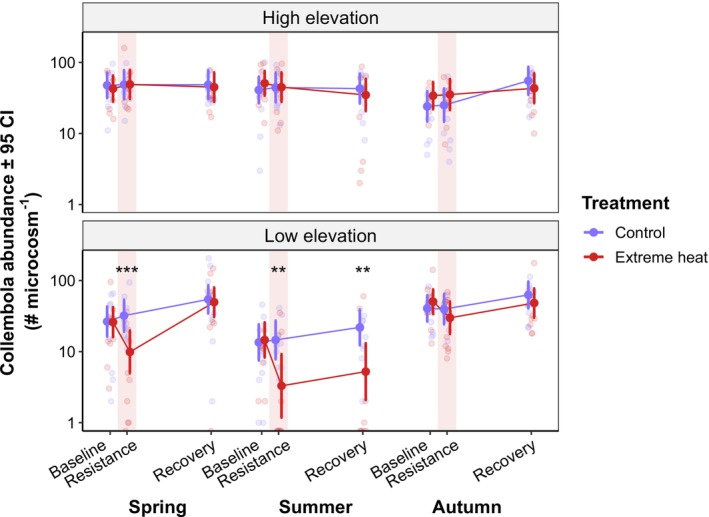
Responses of Collembola abundance to experimental extreme heat events across elevations and at different seasons. Estimated marginal means (±95 confidence intervals) of Collembola abundance (log‐transformed) are shown over the course of the experiments in spring, summer and autumn. The labels on the *x*‐axis specify the different time points in which Collembola densities were assessed during the experiment (i.e., harvests): Baseline (harvest 1); resistance phase (harvest 2); recovery phase (harvest 3). The faded red areas represent the 1‐week extreme heat events. Colours indicate different experimental temperature treatments: Blue: Control; red: Extreme heat. Asterisks show significant differences between treatments at each harvest: **p < 0.01, ***p < 0.001. Full model outputs are provided in Table S9.

Collembola abundance and diversity were affected by extreme heat at low elevation in spring and summer, while the effects in autumn and at high elevation (across seasons) were negligible (Figure 2; Figure S6). At low elevation sites, Collembola abundance declined in spring (mean percentage change ± SE: −69% ± 13%) and summer (−77% ± 14%) at the resistance phase. Remarkably, Collembola abundance at low elevation recovered completely in spring, but significant deviations from control treatments (i.e., negative recovery) persisted in summer (−76% ± 13%; Figure 2, Table S8). Diversity metrics mirrored the responses of Collembola abundance in spring at low elevation (i.e., negative resistance in all diversity metrics, e.g., −49% ± 20% Shannon–Hill; followed by complete recovery), but not in summer, since diversity metrics were not affected by extreme heat in this case (Figure S6).

### 3.2 Collembola Communities: Species‐Specific Abundance Responses

**FIGURE 3:**
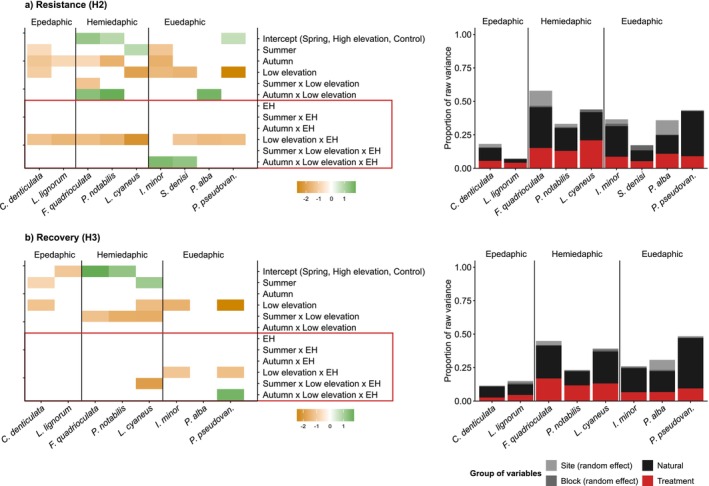
Output of the joint species distribution models (jSDMs) fitted to investigate the responses of Collembola species abundances. We tested the effects of season, elevation, treatment, and their three‐way interactions, in the resistance (a; harvest 2: H2; panels above) and the recovery response (b; harvest 3: H3; panels below). The results from the baseline response are provided in Figure S7. Estimates from the beta parameters (left panels) show the responses of species abundances (*x*‐axis) to each of the model parameters (*y*‐axis). Green and orange colours indicate positive and negative responses with 95% posterior probability, respectively, while blank spaces denote responses that lacked statistical support (should, therefore, be interpreted as neutral response). Species abundances at the intercept (spring, high elevation, control treatment) denote more abundant species in green, less abundant species in orange and blank spaces indicating intermediate abundances (Table S6). Parameters enclosed within the red area represent species responses to the experimental treatment (extreme heat: EH; see Table S6 for an ecological interpretation of the model parameters). The proportion of raw explained variance (right panels) is provided for different groups of variables: Random effects (site and block), natural variables (season and elevation) and treatment (containing the variance explained by all parameters influenced by extreme heat, shown within the red area of the left panels). Collembola species are ordered according to their vertical stratification across the soil profile: Epedaphic (surface‐living), hemi‐edaphic (living in litter and shallow soil layers) and euedaphic (permanently living in the soil).

Out of the nine Collembola species included in the analysis of species abundances (see Methods for the inclusion criteria), eight species showed negative resistance responses in spring at low elevation (Figure 3a). Later, most of them attained a complete recovery (6 out of 9), except for *Protaphorura pseudovanderdrifti*, *Isotomiella minor* and *Lepidocyrtus cyaneus* (Figure 3b). Even though these species occurred at both elevations, they were significantly lesser abundant at low elevation sites (Figure 3, Figure S7). Besides, we found that the vertical stratification across the soil profile of Collembola species did not explain changes in species abundances driven by extreme heat (Figure S8).

### 3.3 Fungal Communities

**FIGURE 4:**
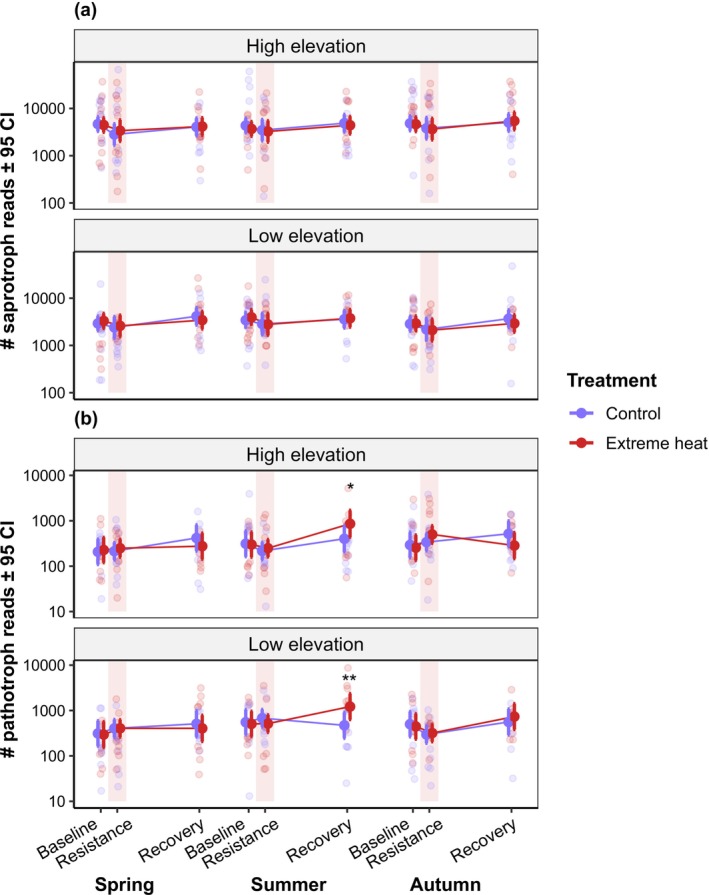
Responses of relative abundances of saprotrophic and pathogenic fungi to experimental extreme heat events across elevations and at different seasons. Estimated marginal means (±95 confidence intervals) of the number of reads (log‐transformed) of saprotrophs (a; upper panel) and pathogenic fungi (b; lower panel) over the course of the experiments in spring, summer and autumn. The labels on the *x*‐axis specify the different time points in which fungal metabarcoding reads were assessed during the experiment (i.e., harvests): Baseline (harvest 1); resistance phase (harvest 2); recovery phase (harvest 3). The faded red areas represent the 1‐week extreme heat events. Colours indicate different experimental temperature treatments: Blue: Control; red: Extreme heat. Stars show significant differences between treatments at each harvest: *p < 0.05, **p < 0.01. Full model outputs are provided in Tables S9 and S10.

The structure of fungal communities remained stable in response to the extreme heat events across elevations and seasons, as extreme heat did not alter either fungal diversity (Figure S9) or, in general terms, the occurrences and relative abundances of fungal species (Figures S10–S12). However, certain fungal trophic groups responded to extreme heat in the recovery response: relative abundances of pathogens increased markedly in summer both at low (+159% ± 123%) and high elevations (+114% ± 104%) (Figure 4b, Table S10). Besides, relative abundances of unassigned fungi increased (+43% ± 53%) in spring at low elevation, as well as those of symbiotic fungi (+144% ± 105%) in summer at high elevation (Figure S13). Conversely, symbiotic fungi declined at the resistance response (−65% ± 17%) in spring at high elevation (Figure S13). The occurrences of several pathogens exposed to extreme heat were higher at the recovery response (mainly in autumn at high elevation; Figure S14), but not their species relative abundances (Figure S12).

### 3.4 Collembola‐Fungal Association Networks at the Recovery Response

**FIGURE 5:**
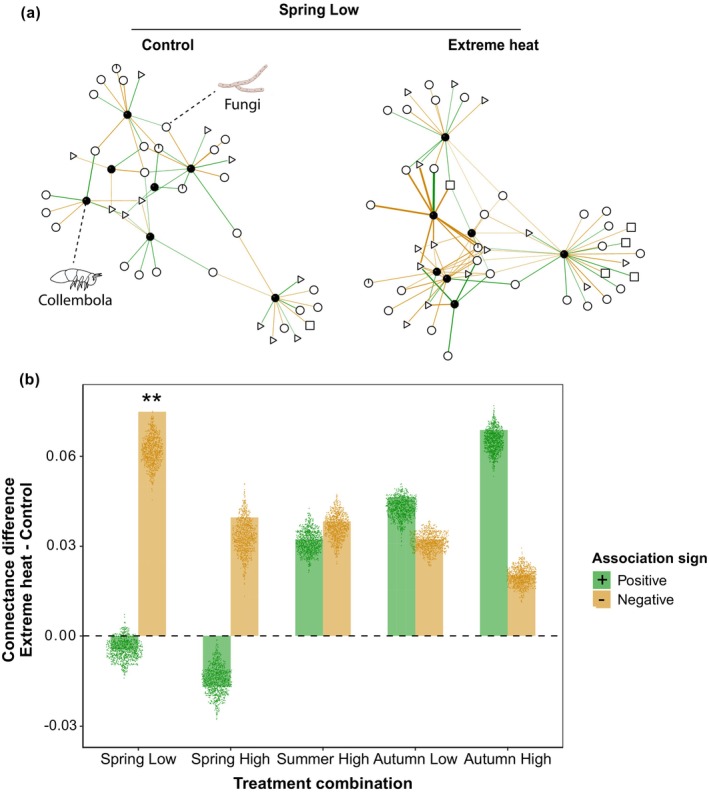
Collembola‐fungal association networks and connectance at the recovery response. (a) Comparison of Collembola‐fungal association networks between control and extreme heat treatments. An example is shown from the association networks from spring at low elevation. Positive links are displayed with green colours and negative links are shown with orange colours. The width of the links is proportional to the strength of the associations (i.e., parameter estimates of the Collembola‐fungal jSDM). Black and white nodes denote Collembola and fungal species, respectively. Different node shapes represent various fungal trophic groups: Saprotrophs (circle), pathogens (square), symbionts (pie) and unassigned fungi (triangle). Nodes without associations (i.e., degree = 0) are not displayed. (b) The differences in connectance between extreme heat and control treatments were calculated and tested against those differences obtained from null models. The height of the barplot shows the observed connectance differences, while the points display the connectance differences from the null models. Positive values indicate higher connectance in extreme heat treatments, whereas negative values denote higher connectance in control treatments. *Z*‐scores and *p*‐values are provided in Table S11. Stars show significant greater observed connectance differences between treatments compared to networks generated from the null models: **p < 0.01. All association networks are shown in Figure S15.

Extreme heat altered the connectance of Collembola‐fungal association networks in recovering lowland communities in spring (Figure 5, Table S11). Compared to random expectations from null models, the connectance of negative associations increased in heat‐exposed networks (connectance difference: 0.075; *p* = 0.004) (Figure 5, Table S11). This rise in network connectance was driven by a higher number of negative associations between Collembola and saprotrophic fungi species (Table S11).

## 4 Discussion

We found that belowground communities responded differently to experimental extreme heat events across elevations and seasons, as well as depending on the trophic level. Collembolan communities were especially susceptible to extreme heat events at low elevations, confirming our initial expectations. However, recovery was season‐dependent at low elevations, as collembolan communities managed to compensate for previous heat‐induced declines in spring but not in summer, further suggesting that collembolans from summer soils in lowlands were the most vulnerable ones. Fungal communities were generally stable to extreme heat events, with some marked exceptions for fungal pathogen species. Our results further revealed that extreme heat altered the structure of Collembola‐fungal associations in recovering lowland communities, mainly by increasing the connectance of negative associations in spring.

### 4.1 Extreme Heat Events Caused Stronger Ecological Effects on Low Elevation Communities

Low elevation belowground communities were disproportionally impacted by extreme heat compared to those at high elevation, particularly the collembolan communities. This finding supports the known geographic patterns of thermal vulnerability across latitudinal gradients (Louthan et al. 2021), demonstrating that organisms currently experiencing warm conditions or occasional hot periods (e.g., at low elevations) are prone to greater physiological costs with further warming (Deutsch et al. 2008; Dillon et al. 2010; Kingsolver et al. 2013). In turn, organisms at high elevations tend to have wider thermal safety limits because their heat tolerances remain constant across elevations (Sunday et al. 2014). Even though the abundances of Collembola at higher elevations remained unaltered by extreme heat, some common highland species were particularly impacted when they also occurred at lower elevations, especially at the recovery response (e.g., *Protaphorura pseudovanderdrifti*, *Lepidocyrtus cyaneus* ; Figure 3). Such negative recovery responses are likely explained by the deleterious impacts of heat on fecundity, as previously shown in laboratory populations of *P. pseudovanderdrifti* (Martínez‐De León et al. 2024). These findings suggest the (elevational) range contraction of typical high‐elevation species in response to extreme heat events, especially as warm‐adapted species may recover better and therefore exclude other species closer to their thermal niche limits (Moore et al. 2023). Importantly, heat extremes of similar severity to those simulated in our experiment are already taking place occasionally (Table S4), underscoring the relevance of our findings for natural communities in the face of present‐day and future heat extremes.

We also found that fungal communities remained generally unaltered in response to the experimental heat events. It has been previously shown that many soil fungal communities are generally robust to extreme heat and drought (Bei et al. 2023; Knight et al. 2024; de Vries et al. 2018), partly because water and nutrients can be redistributed from different parts of the fungal mycelium (Guhr et al. 2015). Nonetheless, certain trophic groups, predominantly pathogens, reacted strongly to the extreme heat events, mainly in the recovery response. In particular, the relative abundances of fungal pathogens increased markedly after exposure to heat events in summer, both at low and at high elevations (Figure 4b, Figure S12, Table S10), corroborating previous findings that hotter conditions promote fungal pathogens at the global scale (Delgado‐Baquerizo, Guerra, et al. 2020). However, given the compositional nature of metabarcoding data, it is unclear whether absolute fungal abundances shifted due to exposure to extreme heat, as has been shown previously in response to long‐term experimental warming (DeAngelis et al. 2015).

### 4.2 Seasonal‐Dependent Effects of Extreme Heat on Low Elevation Communities

Extreme heat events had distinct effects on low elevation collembolan communities depending on whether they occurred in spring or summer. In these seasons, extreme heat generally affected collembolan survival, as revealed by their negative resistance responses. Remarkably, this was followed by a complete recovery of the abundances of most species in spring, indicating that their recruitment managed to compensate for the previous heat‐induced mortality. Those individuals that survived the heat event may have benefited from reduced competition, allowing for a higher fecundity and/or enhanced juvenile viability during the recovery period. By contrast, recovery remained incomplete in the summer season. We suspect that most species used a strategy of seasonal escape (Kefford et al. 2022), which implies that recruitment was possibly delayed until the end of a summer diapause period (Masaki 1980; Testerink 1983). The influence of pathogens might additionally explain the limited recovery of Collembola in summer, given that pathogenic fungi became more abundant in heat‐exposed soils (Figure 4), and were therefore more likely to infect Collembola hosts (Anslan et al. 2018). However, this possibility remains unclear, given that Collembola can exhibit high tolerance to various entomopathogenic fungi found in soils (Dromph and Vestergaard 2002).

In autumn, responses to extreme heat events were generally negligible, or even positive in some Collembola species (Figure 3). As opposed to spring and summer, heat responses in autumn are likely delayed for a much longer period than the recovery phase used in our study. Many species reduce their activity before the onset of winter (Testerink 1983), especially at high elevations. During this period, non‐active individuals need to endure metabolic costs that become even greater during heat events, leading to reduced survival after the winter diapause (Nielsen et al. 2022). It is thus plausible that our recovery responses could not capture the deleterious effects of autumn extreme heat events, which would require the measurement of post‐winter or multiyear effects in controlled experiments (Cope et al. 2023).

### 4.3 Extreme Heat Increased the Connectance of Collembola‐Fungal Association Networks

We show that extreme heat events increased negative associations between Collembola and fungi in recovering communities at low elevation, particularly in spring. While these associations reflect statistical relationships rather than direct feeding interactions, the shifts in network properties may impact soil community functioning under extreme heat. Network alterations persisted at the recovery response, likely due to community restructuring after heat events (Figure 5). Our results suggest that locally abundant saprotrophic fungi, possibly representing poor‐quality resources, hindered Collembola species' recovery, increasing negative Collembola‐fungal associations (Table S11) –even as saprotrophs remained stable. These findings confirm our hypothesis of increased heat‐induced connectance of negative associations, though we did not observe the anticipated increase in positive associations. We suggest that temperature effects on feeding rates (Dell et al. 2011) may cause collembolans to develop more generalised interactions with fungi (Petchey et al. 2010), especially in cooler settings like higher elevations. Further studies examining food web responses during and after heat events, as done in freshwater systems (Polazzo et al. 2023), are needed to verify this expectation in belowground communities.

### 4.4 Key Limitations

We highlight three key limitations of our experiment. First, our experimental design, which used field‐collected soil microcosms incubated under controlled laboratory conditions, enabled us to isolate and assess the effects of spatiotemporal contexts on community responses to extreme heat in a standardised manner; however, this approach inevitably omits key field‐based factors –such as aboveground‐belowground interactions and natural nutrient fluxes‐ that shape soil community dynamics in situ. These ecological processes may modulate recovery trajectories following extreme events and are not fully captured in laboratory settings. As such, while our findings offer important mechanistic insights, they should be extrapolated to natural communities with caution. Second, the number of sites included in our study was two replicated sites at each elevation. The limited spatial contexts present a caveat for any widespread generalisation of our findings, which would require further evaluation of ecological responses to heat extremes in other elevational gradients and seasonal contexts. Third, our experimental setup provided reduced possibilities for behavioural thermoregulation, possibly causing greater responsiveness in certain collembolan communities (Sunday et al. 2014). Soil invertebrates such as Collembola can migrate towards deeper and cooler soil layers (e.g., deeper than 5 cm; i.e., depth of the experimental microcosms) to temporarily avoid heat and drought stress (Holmstrup and Bayley 2013). Even so, migrating individuals could be poorly adapted to the changing environmental conditions encountered in deeper soil depths (e.g., reduced resource availability, increased competition due to aggregation of individuals), eventually leading to population declines in response to prolonged heat events (Sanders et al. 2024).

## 5 Conclusions

Our comparative experiment shows that extreme heat has a stronger impact on lowland communities, especially on invertebrate consumers (Collembola) compared to their microbial resources (fungi), supporting the idea of a trophic mismatch. Notably, collembolan communities managed to recover in spring but not in summer, which emphasises the importance of phenological processes in determining recovery after pulse disturbances like heat extremes. Despite the general stability of fungal communities, heat‐induced shifts in the relative abundances of certain trophic groups could have cascading effects on other ecological processes (e.g., infection prevalence, decomposition of organic matter), especially if these changes prevail over longer timescales. Conversely, ecological and evolutionary changes could help to dampen heat‐induced trophic mismatches, for instance if heat‐tolerant consumer species become more dominant in the communities. Our study illustrates how depicting resistance and recovery to heat extremes in different spatiotemporal contexts (e.g., elevation and seasons) and across trophic groups can contribute to draw a more complete picture of ecological stability in a changing world.

## Author Contributions

Gerard Martínez‐De León and Madhav P. Thakur conceived the study. Gerard Martínez‐De León led the experiments and collected the data, with technical support from Ludovico Formenti. Jörg‐Alfred Salamon conducted the taxonomic determination of Collembola species. Gerard Martínez‐De León analysed the data with the inputs from Madhav P. Thakur. Gerard Martínez‐De León wrote the manuscript with substantial contributions from Madhav P. Thakur. All authors revised and approved the final manuscript.

## Peer Review

The peer review history for this article is available at https://www.webofscience.com/api/gateway/wos/peer‐review/10.1111/ele.70225.

## Supporting information


**Data S1:** ele70225‐sup‐0001‐DataS1.docx.

## Data Availability

The complete dataset and R scripts used in this study are available in the Figshare repository: https://doi.org/10.6084/m9.figshare.26142490. The sequencing data have been deposited in the European Nucleotide Archive (ENA) at EMBL‐EBI under accession number PRJEB96258 (https://www.ebi.ac.uk/ena/browser/view/PRJEB96258).
