# Supplementary material for: Belowground Communities in Lowlands Are Less Stable to Heat Extremes Across Seasons
Source: Ecol Lett. 2025 Oct 3;28(10):e70225. doi: 10.1111/ele.70225 (PMC12493265; doi:10.1111/ele.70225)
Supplement: Supplementary file 1 — Data S1: ele70225‐sup‐0001‐DataS1.docx. [file ELE-28-0-s001.docx]

**Supporting Information for**

Belowground communities in lowlands are less stable to heat extremes across seasons

Table of contents

| Supplementary Methods 1 | Experiment setup |
| --- | --- |
| Supplementary Methods 2 | Fungal ITS metabarcoding |
| Supplementary Methods 3 | Collembola-fungi association networks |
| Table S1 | Description of the field sites |
| Table S2 | Description of soil physicochemical parameters |
| Table S3 | Description of the experimental temperature regimes |
| Table S4 | Comparison between experimental and field-recorded temperatures |
| Table S5 | Collembola species abundances and vertical stratification |
| Table S6 | Ecological interpretation of the parameters from the joint species distribution models |
| Table S7 | Scale reduction factors of the joint species distribution models |
| Table S8 | Output of GLMMs of total Collembola abundance |
| Table S9 | Output of GLMMs of saprotrophic fungi reads |
| Table S10 | Output of GLMMs of pathogenic fungi reads |
| Table S11 | Output of Collembola-fungal association network analysis |
| Table S12 | List of controls used in the amplicon sequencing |
| Fig. S1 | Map of the study area |
| Fig. S2 | Pictures of the field sites |
| Fig. S3 | Daily soil temperatures in the field sites during the study period |
| Fig. S4 | Gravimetric soil water content at field sampling |
| Fig. S5 | Visualization of Collembola and fungal communities |
| Fig. S6 | Diversity profiles of Collembola communities |
| Fig. S7 | Estimates of the Beta parameters of Collembola jSDMs: Baseline |
| Fig. S8 | Estimates of the Gamma parameters of jSDMs: Collembola vertical stratification |
| Fig. S9 | Diversity profiles of fungal communities |
| Fig. S10 | Estimates of the Beta parameters of fungal jSDMs: Baseline |
| Fig. S11 | Estimates of the Beta parameters of fungal jSDMs: Resistance |
| Fig. S12 | Estimates of the Beta parameters of fungal jSDMs: Recovery |
| Fig. S13 | Abundance responses of unassigned and symbiotic fungi |
| Fig. S14 | Estimates of the Gamma parameters of jSDMs: Fungal trophic groups (presence-absence model) |
| Fig. S15 | Association networks between Collembola and fungi |

**Supplementary Methods 1: Experiment setup**

Before all soil cores were sampled, we cut the vegetation at 5 cm from the ground level to avoid overcrowding when soil cores were later incubated in the laboratory. The pots used to contain the soil cores were made of polypropylene (height: 7.5 cm and diameter: 8 cm) with a 90 µm mesh at the bottom and a 5 cm high plastic fence (from the top of the pot), to minimize the escape of invertebrates from the pots while allowing for vegetation growth. The gravimetric soil water content at the time of sampling was determined by drying five additional soil samples at 70 °C for 48h (Table S2; Fig. S3). In order to avoid keeping exceedingly dry soil conditions during the experiments, we made sure that the sampling of soil cores took place shortly after the occurrence of precipitation events in the field sites (> 5 mm during the previous week). Additionally, we took three soil cores across seasons to determine soil pH (Table S2), and one soil core to monitor soil temperature in the incubators over the course of the experiments (collected at a random location within the plots). The incubators (*N* = 6) were randomly rotated among treatments at each season (Table S3).

**Supplementary Methods 2: Fungal ITS metabarcoding**

Prior to amplification of fungal ITS1-2 regions with barcoded primers, the starting DNA was assessed for quantity, quality and purity. We then carried out PCR-amplification targeting the primers ‘TCCGTAGGTGAACCTGC’ (forward) and ‘GCATATCAATAAGCGGAGGA’ (reverse). W then sequenced the full ITS region (ITS1-ITS2) with PacBio Sequel II instrument (Pacific Biosciences, USA). Libraries were loaded into three SMRTcells, each including five blanks and five controls (listed in Table S12). Briefly, after demultiplexing, low-quality reads (<Q20) were discarded, primers trimmed (mean read length after processing: 670 bp), and denoised ASVs were obtained. Next, singletons and ASVs with less than five reads were filtered out, and taxonomical assignment with VSEARCH was performed using the UNITE QIIME release 9 (Abarenkov *et al.* 2023). We then merged the data from the different sequencing runs and retained only fungal ASVs agglomerated at the species level (R package phyloseq) (McMurdie & Holmes 2013). We then obtained the main trophic strategy of each fungal genus (i.e., saprotroph, symbiotroph, pathogenic) from the FungalTraits database (Põlme *et al.* 2020). To do so, we merged all primary lifestyle categories related, respectively, to saprotrophic modes (e.g., soil and litter saprotrophs), symbiotrophic (including mycorrhizal and lichenized fungi), and pathogenic modes (including parasites, endophytes, epiphytes and sooty molds).

**Supplementary Methods 3: Collembola-fungi association networks**.

We estimated associations between Collembola and fungi using joint species distribution models, followed by the analysis of network properties (i.e, connectance) to summarize these associations at the network level. This analysis was restricted to the recovery response, to gain more robust and ecologically meaningful insights into the role of biotic effects in mediating responses to extreme heat. Resistance responses are primarily driven by abiotic effects of extreme heat on species’ abundances, while recovery responses can be more strongly influenced by biotic effects, such as associations with other species (Martínez-De León & Thakur, 2024). This is because heat-driven changes in the abundance of one species (e.g., fungi) may take time to affect the abundance of a second species (e.g., Collembola). We assume that our measurement of recovery (i.e., five weeks after the end of the extreme heat events) can generally capture such a time lag in disturbance effects across the two trophic levels (Jackson et al., 2021). For this analysis, we created separate subsets from the full dataset for each elevation and season, resulting in six subsets, each containing 20 samples. We applied a prevalence threshold of 25% within each subset (i.e., discarding species occurring in fewer than five samples) for all Collembola and fungal species, as previously described. Due to the very low prevalence of Collembola species in summer at low elevation, we could not determine associations in this case. Next, we built the jSDMs using fungal species relative abundances as response variables (log-transformed and scaled abundances, conditional on presence), while treating Collembola species abundances (log-transformed +1 and scaled) and their interactive effects with extreme heat as explanatory variables. We retained the associations between Collembola and fungi with 95% credible intervals not overlapping zero for control and extreme heat treatments, using the ci function from the bayestestR package (Makowski et al., 2019). Extreme heat associations were obtained by summing the parameter estimates of every Collembola-fungal association in the control treatment and the interactive effects of extreme heat, in all posterior samples. These associations can be indicative of bottom-up regulation through feeding (positive associations) or repulsion (negative associations), but they should be interpreted with care, as they may also capture the signal of joint responses to unmeasured abiotic variables (Blanchet et al., 2020; Ovaskainen et al., 2017). Additionally, the mismatch in the spatial scales at which Collembola and fungi were measured (see Data collection in Methods) may lessen the statistical signal of their associations (Blanchet et al., 2020), particularly due to small-scale variation in fungal composition within the soil cores (Erktan et al., 2020) (although experimental replication partly accounts for this issue; see Fig. S5).

After fitting the jSDMs, we examined how two association network properties differed between control and extreme heat treatments: connectance and network dissimilarity. We visualized the associations resulting from the Collembola-fungi jSDMs using the igraph package (Csárdi et al., 2024). For the analysis of connectance (i.e., the ratio of the number of realized associations to the number of potential associations) (May, 1972, p. 197), we calculated the observed differences in network connectance between the experimental treatments, and further generated null models to test how the observed differences diverged from random expectations. To do this, we first trimmed the control and extreme heat networks obtained from the same jSDM (i.e., same spatiotemporal context) to retain only the species having associations in either of the two networks (metaweb). We then produced 1000 permutations of each association network using the r2dtable algorithm (implemented in the package vegan) (Oksanen et al., 2022), as this method keeps the matrix dimensions and marginal totals constant while allowing for variation in the number of non-zero elements (i.e., number of Collembola-fungal associations), and hence connectance (Dormann et al., 2009). We then calculated differences in connectance between the random networks from control and extreme heat treatments, and compared these to the observed differences. To do so, we computed z-scores (Eq. 1), and obtained the corresponding p-values using two-tailed tests of population proportion.

Eq. 1 $z=\frac{Observed connectance difference-Mean null connectance differences}{SD null connectance differences}$

Finally, to pinpoint the specific fungal groups driving changes in network connectance, we repeated the connectance analysis separately for saprotrophic and pathogenic fungi.

**Table S1**. Description of the field sites. All plots were located in extensively managed dry meadows (i.e. one hay cut per year occurring not before July 1^st^ and/or low-intensity grazing, no inputs of fertilizer or irrigation), with no recent soil disturbances.

| **Location** | **Chasseral** | **Le Landeron** | **Chasseron** | **Onnens** |
| --- | --- | --- | --- | --- |
| **Block** | North | North | South | South |
| **Elevation** | High (1558 m) | Low (481 m) | High (1565 m) | Low (540 m) |
| **Coordinates** | 47°07’43” N 7°02’52” E | 47°03’39” N  7°03’49” E | 46°50’58” N 6°32’18? E | 46°50’49” N 6°41’07” E |
| **Aspect** | 170° (S) | 210° (SSW) | 190° (S) | 140° (SE) |
| **Slope** | 6% | 21% | 10% | 5% |
| **Mowing (frequency, period)** | Annually; August-September | Biannually; July-August | No mowing | Annually; July-August |
| **Grazing (type, period)** | Not grazed | Not grazed | Cow grazing in the past years, currently not grazed | Sheep grazing, October-November |
| **Dominant plant species** | *Carex nigra*, *Agrostis capillaris*, *Dactylis glomerata* | *Securigera varia, Bromus erectus, Carex* sp. | *Carex montana, Sanguisorba officinalis, Agrostis capillaris* | *Bromus erectus*, *Trisetum flavescens*,  *Salvia pratensis* |

| **Site**  **(block and elevation)** | **Season** | **Soil pH** | **Bulk density (g cm^-3^)** | **Gravimetric water content (%)** |
| --- | --- | --- | --- | --- |
| Chasseral (north high) | Spring | 5.54 ± 0.67 | 0.60 ± 0.15 | 44.91 ± 3.94 |
|  | Summer |  | 0.69 ± 0.11 | 36.10 ± 2.37 |
|  | Autumn |  | 0.80 ± 0.20 | 36.70 ± 2.60 |
| Le Landeron (north low) | Spring | 7.91 ± 0.05 | 0.84 ± 0.13 | 24.47 ± 3.90 |
|  | Summer |  | 0.95 ± 0.21 | 24.51 ± 1.93 |
|  | Autumn |  | 0.89 ± 0.14 | 22.44 ± 1.07 |
| Chasseron (south high) | Spring | 5.10 ± 0.21 | 0.72 ± 0.12 | 44.00 ± 3.09 |
|  | Summer |  | 0.68 ± 0.13 | 30.35 ± 2.86 |
|  | Autumn |  | 0.61 ± 0.13 | 28.90 ± 4.55 |
| Onnens (south low) | Spring | 5.98 ± 0.25 | 1.19 ± 0.15 | 25.56 ± 1.30 |
|  | Summer |  | 1.27 ± 0.05 | 15.29 ± 1.10 |
|  | Autumn |  | 1.29 ± 0.16 | 20.35 ± 1.38 |

**Table S2.** Description of soil physicochemical parameters at the time of field sampling (i.e., not exposed to subsequent incubation in the laboratory) across the three studied seasons (spring, summer, autumn). For soil pH, we measured *N =* 3 per site and across seasons. For bulk density and gravimetric water content, we measured *N* = 5 per each site and season.

**Table S3**. Description of the experimental temperature regimes. Climatic data representative of high elevations was obtained from the weather station in Chasseral (47°07′54″N 7°03′16″E; 1596 m.a.s.l.), whereas for low elevation, we acquired data from the weather station in Neuchâtel (47°00′00″N 6°57′12″E; 485 m.a.s.l.). We retrieved air temperatures recorded at 2 m aboveground from the period 2015-2020 (source: Meteoswiss). The climate in the study area is temperate continental, with low elevations characterized by average yearly temperatures of 10.7 °C (monthly average of the coldest and warmest month: 1.8 °C and 20.1 °C, respectively) and 956 mm of annual precipitation. At high elevations, the average yearly temperature is 4.3 °C (monthly average of the coldest and warmest months: −2.8 °C and 12.1 °C, respectively) with 1396 mm of annual precipitation. Control temperatures were set as the average daily temperature over the reference period per elevation and season. To establish the extreme heat events for each elevation and season, we adopted the 99^th^ percentile of daily temperatures across the reference period for spring (May-June), summer (July-August) and autumn (Spring-October). For both control and extreme heat temperature regimes, we included a diel light and temperature cycle (8h night/ 16h day), with a 6 °C-amplitude between night and day. C: Control temperature, EH: Extreme heat. The identity of the incubators (#1 to #4) containing each treatment combination is provided.

| **Elevation** | **Season** | **Temperature treatment** | **Average daily temperature (°C)** | **Daytime temperature (°C)** | **Nighttime temperature (°C)** | **Incubator ID** |
| --- | --- | --- | --- | --- | --- | --- |
| High | Spring | C | 8.8 | 10.8 | 4.8 | #3 |
|  |  | EH | 20.5 | 22.5 | 16.5 | #1 |
|  | Summer | C | 13.5 | 15.5 | 9.5 | #1 |
|  |  | EH | 21.7 | 23.7 | 17.7 | #4 |
|  | Autumn | C | 7.3 | 9.3 | 3.3 | #2 |
|  |  | EH | 16.2 | 18.2 | 12.2 | #4 |
| Low | Spring | C | 16.5 | 18.5 | 12.5 | #2 |
|  |  | EH | 26.6 | 28.6 | 22.6 | #4 |
|  | Summer | C | 21.2 | 23.2 | 17.2 | #2 |
|  |  | EH | 28.0 | 30.0 | 24.0 | #3 |
|  | Autumn | C | 13.6 | 15.6 | 9.6 | #3 |
|  |  | EH | 21.7 | 23.7 | 17.7 | #1 |

**Table S4**. Output of model used to compare the average daily soil temperature (measured at 3-5 cm depth) in the extreme heat events simulated in the lab, against the hottest days recorded in the field sites during the study period (*N* = 6 days, per each elevation and season combination). This analysis was conducted to evaluate the severity of our experimental treatments compared to the natural variability of heat extremes in the field sites. We fitted a linear mixed effect model with the R package nlme v.3.1-163 (Pinheiro et al., 2023), accounting for heterogeneity of residuals by taking the origin of the data (field or lab) as an offset term, due to the greater variance of the data collected from the field compared to the temperature data from the lab experiments. We note that the year in which the study took place (2022) was one of the warmest on record in the area, exceeding the norm of monthly mean temperature of May-October by 2.3-2.5 °C on average (relative to the 1990-2010 reference period; source Meteoswiss).

| Average daily soil temperature (°C) | | | | | | |
| --- | --- | --- | --- | --- | --- | --- |
| Elevation | Season | Origin of data | Estimate | SE | *P* | Marginal/  Conditional R^2^ |
|  |  |  |  |  |  |  |
| High | Spring | Lab | 19.60 | 0.33 | 0.060 | 0.993 / 0.998 |
|  |  | Field | 18.02 | 0.24 |  |  |
|  | Summer | Lab | 20.75 | 0.33 | 0.087 |  |
|  |  | Field | 19.44 | 0.26 |  |  |
|  | Autumn | Lab | 15.72 | 0.33 | **0.011** |  |
|  |  | Field | 11.87 | 0.26 |  |  |
| Low | Spring | Lab | 25.47 | 0.33 | 0.962 |  |
|  |  | Field | 25.49 | 0.24 |  |  |
|  | Summer | Lab | 27.23 | 0.33 | 0.322 |  |
|  |  | Field | 26.69 | 0.26 |  |  |
|  | Autumn | Lab | 21.03 | 0.33 | **0.010** |  |
|  |  | Field | 16.82 | 0.26 |  |  |

**Table S5.** Total Collembola species abundances (*N* = 360) and vertical stratification of the species across the soil profile: epedaphic (surface-living), hemiedaphic (living in litter and upper soil layers) and euedaphic (permanently living in the soil). The sources for the identification of Collembola species were: Dunger & Schlitt (2011); Fjellberg (1998, 2007); Gisin (1960); Hopkin (2007); Thibaud *et al.* (2004). The vertical stratification of each Collembola species was extracted mainly from Gisin (1943), as well as Chauvat *et al.* (2014); Ferlian *et al.* (2015); Leinaas & Bleken (1983); Urbášek & Rusek (1994). The abundances of immature individuals that could not be assigned to a particular species are displayed at the bottom of the table.

| **Collembola species** | **Family** | **Vertical stratification** | **Total abundance** |
| --- | --- | --- | --- |
| *Folsomia quadrioculata* | Isotomidae | Hemiedaphic | 3502 |
| *Parisotoma notabilis* | Isotomidae | Hemiedaphic | 2867 |
| *Isotoma viridis* | Isotomidae | Hemiedaphic | 918 |
| *Isotomiella minor* | Isotomidae | Euedaphic | 890 |
| *Protaphorura pseudovanderdrifti* | Onychiuridae | Euedaphic | 863 |
| *Lepidocyrtus cyaneus* | Entomobryidae | Hemiedaphic | 820 |
| *Pseudosinella alba* | Entomobryidae | Euedaphic | 750 |
| *Lepidocyrtus lignorum* | Entomobryidae | Epedaphic | 461 |
| *Ceratophysella denticulata* | Hypogastruridae | Epedaphic | 351 |
| *Stenaphorura denisi* | Tullbergiidae | Euedaphic | 288 |
| *Sminthurinus signatus* | Katiannidae | Hemiedaphic | 251 |
| *Choreutinula inermis* | Hypogastruridae | - | 116 |
| *Sminthurinus aureus* | Katiannidae | Epedaphic | 48 |
| *Sphaeridia pumilis* | Sminthurididae | Hemiedaphic | 41 |
| *Neanura muscorum* | Neanuridae | Hemiedaphic | 20 |
| *Sminthurus viridis* | Sminthurididae | Epedaphic | 15 |
| *Orchesella flavescens* | Orchesellidae | Epedaphic | 5 |
| *Entomobrya multifasciata* | Entomobryidae | Epedaphic | 4 |
| *Pogonognathellus flavescens* | Tomoceridae | Hemiedaphic | 2 |
| *Heteromurus nitidus* | Orchesellidae | Euedaphic | 1 |
| **Immature individuals** |  |  |  |
| Isotomidae |  |  | 397 |
| Hypogastruridae |  |  | 191 |
| Entomobryidae |  |  | 132 |
| Symphypleona |  |  | 7 |

**Table S6.** Ecological interpretation of the parameters from the joint species distribution models (jSDMs) used in our study. We tested the effects of season, elevation, treatment, and their three-way interactions, on Collembola and fungal species relative abundances. In the schematic visualization, green and orange lines represent positive and negative parameter estimates, respectively, while grey lines represent estimates that lack statistical support (i.e., blank fields in Fig. 3).

| **Parameter** | **Ecological interpretation** | **Schematic visualization** |
| --- | --- | --- |
| Intercept | Species abundances in the treatment combination set as the intercept: spring, at high elevation, in the control treatment. | 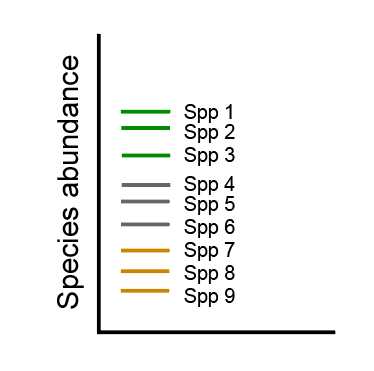 |
| Summer | Shifts in abundance from spring to summer (relative to the intercept). | 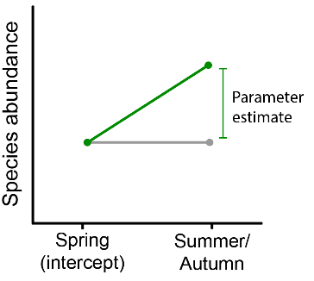 |
| Autumn | Shifts in abundance from spring to autumn (relative to the intercept). |  |
| Low elevation | Shifts in abundance from high to low elevation (relative to the intercept). | 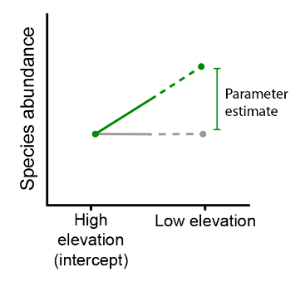 |
| Summer x Low elevation | Given the seasonal abundance shifts as described above, it shows whether this effect is modulated by elevation (in control treatment). | 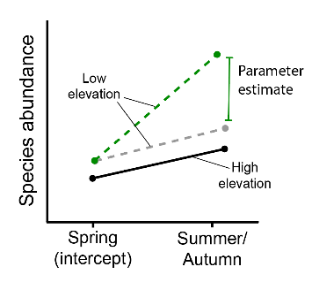 |
| Autumn x Low elevation |  |  |
| EH (extreme heat; including all the interactions involved) | Effect of the extreme heat event, compared to their corresponding reference level in the control treatment. | 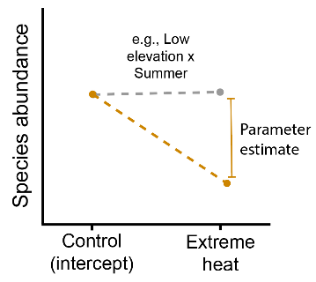­­­­ |

**Table S7.** Potential scale reduction factors for the parameters estimated in the joint species distribution models. The thinning intervals and the number of samples used as burn-in were adjusted for the different models according to the amount required to achieve adequate model convergence (Collembola model: thinning 1,000 and burn-in 125,000; fungal models: thinning 300 and burn-in 37,500; Collembola-fungi association models: thinning 100 and burn-in 12,500).

| **Model** | Collembola | | | | | | | | | |
| --- | --- | --- | --- | --- | --- | --- | --- | --- | --- | --- |
| **Harvest** | Baseline | | | Resistance | | | | Recovery | | |
| **Parameter** | Beta | Gamma | | Beta | | Gamma | | Beta | | Gamma |
| **Min.** | 1.00 | 1.00 | | 1.00 | | 1.00 | | 1.00 | | 1.00 |
| **1^st^ Qu.** | 1.00 | 1.00 | | 1.00 | | 1.00 | | 1.00 | | 1.00 |
| **Median** | 1.00 | 1.00 | | 1.00 | | 1.00 | | 1.00 | | 1.00 |
| **Mean** | 1.00 | 1.00 | | 1.00 | | 1.00 | | 1.00 | | 1.00 |
| **3^rd^ Qu.** | 1.00 | 1.01 | | 1.00 | | 1.00 | | 1.00 | | 1.00 |
| **Max.** | 1.01 | 1.01 | | 1.01 | | 1.01 | | 1.01 | | 1.01 |
|  | | | | | | | | | | |
| **Model** | Fungi (presence-absence) | | | | | | | | | |
| **Harvest** | Baseline | | | Resistance | | | | Recovery | | |
| **Parameter** | Beta | Gamma | | Beta | | Gamma | | Beta | | Gamma |
| **Min.** | 1.00 | 1.00 | | 1.00 | | 1.00 | | 1.00 | | 1.00 |
| **1^st^ Qu.** | 1.00 | 1.00 | | 1.00 | | 1.00 | | 1.00 | | 1.00 |
| **Median** | 1.00 | 1.00 | | 1.00 | | 1.00 | | 1.00 | | 1.00 |
| **Mean** | 1.00 | 1.00 | | 1.00 | | 1.00 | | 1.00 | | 1.00 |
| **3^rd^ Qu.** | 1.00 | 1.00 | | 1.00 | | 1.00 | | 1.00 | | 1.00 |
| **Max.** | 1.01 | 1.01 | | 1.02 | | 1.01 | | 1.01 | | 1.01 |
|  | | | | | | | | | | |
| **Model** | Fungi (abundance conditional on presence) | | | | | | | | | |
| **Harvest** | Baseline | | | Resistance | | | | Recovery | | |
| **Parameter** | Beta | Gamma | | Beta | | Gamma | | Beta | | Gamma |
| **Min.** | 1.00 | 1.00 | | 1.00 | | 1.00 | | 1.00 | | 1.00 |
| **1^st^ Qu.** | 1.00 | 1.00 | | 1.00 | | 1.00 | | 1.00 | | 1.00 |
| **Median** | 1.00 | 1.00 | | 1.00 | | 1.00 | | 1.00 | | 1.00 |
| **Mean** | 1.00 | 1.00 | | 1.00 | | 1.00 | | 1.00 | | 1.00 |
| **3^rd^ Qu.** | 1.00 | 1.00 | | 1.00 | | 1.00 | | 1.00 | | 1.00 |
| **Max.** | 1.01 | 1.01 | | 1.02 | | 1.01 | | 1.01 | | 1.01 |
|  | | | | | | | | | | |
| **Model** | Collembola-fungal models (only recovery) | | | | | | | | | |
| **Parameter** | Beta | | | | | | | | | |
| **Treatment** | Low spring | | High spring | | High summer | | Low autumn | | High autumn | |
| **Min.** | 1.00 | | 1.00 | | 1.00 | | 1.00 | | 1.00 | |
| **1^st^ Qu.** | 1.00 | | 1.00 | | 1.00 | | 1.00 | | 1.00 | |
| **Median** | 1.00 | | 1.00 | | 1.00 | | 1.00 | | 1.00 | |
| **Mean** | 1.00 | | 1.00 | | 1.00 | | 1.00 | | 1.00 | |
| **3^rd^ Qu.** | 1.00 | | 1.00 | | 1.00 | | 1.00 | | 1.00 | |
| **Max.** | 1.02 | | 1.02 | | 1.02 | | 1.08 | | 1.04 | |

**Table S8**. Output of the generalized linear mixed-effects model with negative binomial distribution used to evaluate the effect of the temperature treatments, modulated by elevation and season, on total Collembola abundances. Separate models were fit for each experimental harvest: baseline (harvest 1, before extreme heat), resistance (harvest 2, at the end of extreme heat) and recovery (harvest 3, five weeks after the end of extreme heat). Estimates, standard errors (SE), p-values (*P*) of the contrasts between temperature treatments, marginal and conditional R^2^ (trigamma estimate) are provided (Nakagawa & Schielzeth, 2013). Significant p*-*values (*P* < 0.05) are highlighted in bold. Abbreviations of temperature treatment levels: C: Control temperature, EH: Extreme heat.

| Total Collembola abundances (log-scale) | | | | | | | |
| --- | --- | --- | --- | --- | --- | --- | --- |
|  | Elevation | Season | Temperature treatment | Estimate | SE | *P* | Marginal/  Conditional R^2^ |
|  |  |  |  |  |  |  |  |
| Baseline (harvest 1) | High | Spring | C | 3.86 | 0.21 | 0.651 | 0.263/0.305 |
|  |  |  | EH | 3.76 | 0.21 |  |  |
|  |  | Summer | C | 3.71 | 0.22 | 0.356 |  |
|  |  |  | EH | 3.93 | 0.20 |  |  |
|  |  | Autumn | C | 3.18 | 0.25 | 0.218 |  |
|  |  |  | EH | 3.53 | 0.23 |  |  |
|  | Low | Spring | C | 3.28 | 0.25 | 0.949 |  |
|  |  |  | EH | 3.26 | 0.25 |  |  |
|  |  | Summer | C | 2.60 | 0.30 | 0.827 |  |
|  |  |  | EH | 2.68 | 0.29 |  |  |
|  |  | Autumn | C | 3.70 | 0.22 | 0.356 |  |
|  |  |  | EH | 3.92 | 0.20 |  |  |
| Resistance (harvest 2) | High | Spring | C | 3.88 | 0.24 | 0.969 | 0.512/0.566 |
|  |  |  | EH | 3.89 | 0.24 |  |  |
|  |  | Summer | C | 3.79 | 0.24 | 0.965 |  |
|  |  |  | EH | 3.80 | 0.24 |  |  |
|  |  | Autumn | C | 3.22 | 0.27 | 0.208 |  |
|  |  |  | EH | 3.56 | 0.25 |  |  |
|  | Low | Spring | C | 3.47 | 0.26 | **<0.001** |  |
|  |  |  | EH | 2.29 | 0.35 |  |  |
|  |  | Summer | C | 2.68 | 0.32 | **0.007** |  |
|  |  |  | EH | 1.19 | 0.52 |  |  |
|  |  | Autumn | C | 3.68 | 0.25 | 0.277 |  |
|  |  |  | EH | 3.40 | 0.27 |  |  |
| Recovery (harvest 3) | High | Spring | C | 3.88 | 0.24 | 0.787 | 0.356/0.387 |
|  |  |  | EH | 3.80 | 0.24 |  |  |
|  |  | Summer | C | 3.76 | 0.25 | 0.503 |  |
|  |  |  | EH | 3.55 | 0.26 |  |  |
|  |  | Autumn | C | 4.01 | 0.23 | 0.378 |  |
|  |  |  | EH | 3.77 | 0.24 |  |  |
|  | Low | Spring | C | 4.00 | 0.23 | 0.747 |  |
|  |  |  | EH | 3.91 | 0.24 |  |  |
|  |  | Summer | C | 3.09 | 0.29 | **0.005** |  |
|  |  |  | EH | 1.65 | 0.47 |  |  |
|  |  | Autumn | C | 4.14 | 0.22 | 0.323 |  |
|  |  |  | EH | 3.88 | 0.24 |  |  |

**Table S9.** Output of the generalized linear mixed-effects model with negative binomial distribution used to evaluate the effect of the temperature treatments, modulated by elevation and season, on the number of metabarcoding reads of saprotrophic fungi (representing relative abundances). Separate models were fit for each experimental harvest: baseline (harvest 1, before extreme heat), resistance (harvest 2, at the end of extreme heat) and recovery (harvest 3, five weeks after the end of extreme heat). Estimates, standard errors (SE), p-values (*P*) of the contrasts between temperature treatments, marginal and conditional R^2^ (trigamma estimate) are provided (Nakagawa & Schielzeth, 2013). Significant p*-*values (*P* < 0.05) are highlighted in bold. Abbreviations of temperature treatment levels: C: Control temperature, EH: Extreme heat.

| Number of reads of saprotrophic fungi (log-scale) | | | | | | | |
| --- | --- | --- | --- | --- | --- | --- | --- |
|  | Elevation | Season | Temperature treatment | Estimate | SE | *P* | Marginal/  Conditional R^2^ |
|  |  |  |  |  |  |  |  |
| Baseline (harvest 1) | High | Spring | C | 8.448 | 0.184 | 0.785 | 0.885/0.909 |
|  |  |  | EH | 8.403 | 0.184 |  |  |
|  |  | Summer | C | 8.377 | 0.185 | 0.325 |  |
|  |  |  | EH | 8.212 | 0.184 |  |  |
|  |  | Autumn | C | 8.483 | 0.187 | 0.798 |  |
|  |  |  | EH | 8.440 | 0.185 |  |  |
|  | Low | Spring | C | 7.981 | 0.185 | 0.478 |  |
|  |  |  | EH | 8.101 | 0.190 |  |  |
|  |  | Summer | C | 8.139 | 0.184 | 0.399 |  |
|  |  |  | EH | 8.279 | 0.184 |  |  |
|  |  | Autumn | C | 7.957 | 0.185 | 0.895 |  |
|  |  |  | EH | 7.979 | 0.185 |  |  |
| Resistance (harvest 2) | High | Spring | C | 7.942 | 0.269 | 0.249 | 0.839/0.914 |
|  |  |  | EH | 8.127 | 0.269 |  |  |
|  |  | Summer | C | 8.151 | 0.269 | 0.693 |  |
|  |  |  | EH | 8.088 | 0.269 |  |  |
|  |  | Autumn | C | 8.244 | 0.269 | 0.798 |  |
|  |  |  | EH | 8.203 | 0.270 |  |  |
|  | Low | Spring | C | 7.785 | 0.272 | 0.636 |  |
|  |  |  | EH | 7.862 | 0.269 |  |  |
|  |  | Summer | C | 7.951 | 0.269 | 0.917 |  |
|  |  |  | EH | 7.935 | 0.269 |  |  |
|  |  | Autumn | C | 7.688 | 0.271 | 0.846 |  |
|  |  |  | EH | 7.656 | 0.269 |  |  |
| Recovery (harvest 3) | High | Spring | C | 8.309 | 0.220 | 0.874 | 0.838/0.914 |
|  |  |  | EH | 8.330 | 0.220 |  |  |
|  |  | Summer | C | 8.489 | 0.220 | 0.470 |  |
|  |  |  | EH | 8.392 | 0.220 |  |  |
|  |  | Autumn | C | 8.526 | 0.220 | 0.580 |  |
|  |  |  | EH | 8.601 | 0.221 |  |  |
|  | Low | Spring | C | 8.327 | 0.220 | 0.162 |  |
|  |  |  | EH | 8.140 | 0.220 |  |  |
|  |  | Summer | C | 8.184 | 0.220 | 0.730 |  |
|  |  |  | EH | 8.230 | 0.220 |  |  |
|  |  | Autumn | C | 8.210 | 0.220 | 0.081 |  |
|  |  |  | EH | 7.980 | 0.220 |  |  |

**Table S10.** Output of the generalized linear mixed-effects model with negative binomial distribution used to evaluate the effect of the temperature treatments, modulated by elevation and season, on the number of metabarcoding reads of pathogenic fungi (representing relative abundances). Separate models were fit for each experimental harvest: baseline (harvest 1, before extreme heat), resistance (harvest 2, at the end of extreme heat) and recovery (harvest 3, five weeks after the end of extreme heat). Estimates, standard errors (SE), p-values (*P*) of the contrasts between temperature treatments, marginal and conditional R^2^ (trigamma estimate) are provided. Significant p*-*values (*P* < 0.05) are highlighted in bold. Abbreviations of temperature treatment levels: C: Control temperature, EH: Extreme heat.

| Number of reads of pathogenic fungi (log-scale) | | | | | | | |
| --- | --- | --- | --- | --- | --- | --- | --- |
|  | Elevation | Season | Temperature treatment | Estimate | SE | *P* | Marginal/  Conditional R^2^ |
|  |  |  |  |  |  |  |  |
| Baseline (harvest 1) | High | Spring | C | 5.331 | 0.329 | 0.770 | 0.604/0.664 |
|  |  |  | EH | 5.424 | 0.328 |  |  |
|  |  | Summer | C | 5.743 | 0.336 | 0.899 |  |
|  |  |  | EH | 5.701 | 0.328 |  |  |
|  |  | Autumn | C | 5.681 | 0.331 | 0.654 |  |
|  |  |  | EH | 5.540 | 0.330 |  |  |
|  | Low | Spring | C | 5.745 | 0.330 | 0.855 |  |
|  |  |  | EH | 5.686 | 0.337 |  |  |
|  |  | Summer | C | 6.315 | 0.328 | 0.784 |  |
|  |  |  | EH | 6.228 | 0.328 |  |  |
|  |  | Autumn | C | 6.214 | 0.328 | 0.722 |  |
|  |  |  | EH | 6.100 | 0.331 |  |  |
| Resistance (harvest 2) | High | Spring | C | 5.367 | 0.227 | 0.649 | 0.609/0.609 |
|  |  |  | EH | 5.514 | 0.233 |  |  |
|  |  | Summer | C | 5.374 | 0.227 | 0.662 |  |
|  |  |  | EH | 5.515 | 0.227 |  |  |
|  |  | Autumn | C | 5.825 | 0.228 | 0.223 |  |
|  |  |  | EH | 6.214 | 0.229 |  |  |
|  | Low | Spring | C | 6.002 | 0.231 | 0.995 |  |
|  |  |  | EH | 6.004 | 0.226 |  |  |
|  |  | Summer | C | 6.522 | 0.226 | 0.383 |  |
|  |  |  | EH | 6.242 | 0.227 |  |  |
|  |  | Autumn | C | 5.690 | 0.227 | 0.815 |  |
|  |  |  | EH | 5.764 | 0.226 |  |  |
| Recovery (harvest 3) | High | Spring | C | 6.034 | 0.339 | 0.171 | 0.634/0.703 |
|  |  |  | EH | 5.618 | 0.338 |  |  |
|  |  | Summer | C | 5.995 | 0.340 | **0.024** |  |
|  |  |  | EH | 6.756 | 0.349 |  |  |
|  |  | Autumn | C | 6.246 | 0.336 | 0.053 |  |
|  |  |  | EH | 5.650 | 0.339 |  |  |
|  | Low | Spring | C | 6.232 | 0.338 | 0.470 |  |
|  |  |  | EH | 6.003 | 0.340 |  |  |
|  |  | Summer | C | 6.156 | 0.336 | **0.002** |  |
|  |  |  | EH | 7.107 | 0.338 |  |  |
|  |  | Autumn | C | 6.327 | 0.336 | 0.371 |  |
|  |  |  | EH | 6.599 | 0.335 |  |  |

**Table S11.** Output of the network analysis evaluating differences between the Collembola-fungal association networks from extreme heat and control treatments. Z-scores and p-values were computed to establish whether the observed connectance differences were significantly greater or smaller compared to those from random networks generated with null models (1000 permutations for each network). Significant p-values (*P* < 0.05) are highlighted in bold. The connectance analysis was repeated separately for each fungal trophic group, on subsets of networks only made of either pathogens or saprotrophs. Connectance differences within the subsets of specific fungal groups are indicated; asterisks indicate the level of statistical significance (**P* < 0.05). The observed association networks are displayed in Fig. S12.

| Elevation | Season | Sign of associations | Connectance extreme heat | Connectance control | Connectance difference | z-score | *P* | Connectance differences within fungal groups |
| --- | --- | --- | --- | --- | --- | --- | --- | --- |
| High | Spring | Positive | 0.082 | 0.099 | -0.017 | -0.470 | 0.639 | None |
|  |  | Negative | 0.124 | 0.085 | 0.040 | 1.224 | 0.221 | None |
|  | Summer | Positive | 0.076 | 0.044 | 0.032 | 0.266 | 0.790 | None |
|  |  | Negative | 0.097 | 0.058 | 0.038 | 0.467 | 0.641 | None |
|  | Autumn | Positive | 0.082 | 0.099 | -0.017 | -0.470 | 0.639 | None |
|  |  | Negative | 0.075 | 0.055 | 0.020 | 0.310 | 0.756 | None |
| Low | Spring | Positive | 0.059 | 0.063 | -0.005 | -0.054 | 0.957 | None |
|  |  | Negative | 0.136 | 0.061 | 0.075 | 2.855 | **0.004** | Saprotroph* |
|  | Autumn | Positive | 0.078 | 0.032 | 0.046 | 1.444 | 0.149 | None |
|  |  | Negative | 0.087 | 0.054 | 0.032 | 0.462 | 0.644 | None |

**Table S12.** List of the controls incorporated in the amplicon sequencing pipeline.

| **Type of control** | **Description** | **Reference** |
| --- | --- | --- |
| Blank | Buffers from the extraction kit; added at the extraction phase | <https://www.qiagen.com/us/products/discovery-and-translational-research/dna-rna-purification/dna-purification/microbial-dna/dneasy-powersoil-pro-kit> |
| Negative | Elution buffer: buffer used to dilute samples, primers in MasterMix | [https://www.pacb.com/wp-content/uploads/Procedure-Checklist-%E2%80%93-Amplification-of-Full-Length-16S-Gene-with-Barcoded-Primers-for-Multiplexed-SMRTbell-Library-Preparation-and-Sequencing.pdf](https://eur03.safelinks.protection.outlook.com/?url=https%3A%2F%2Fwww.pacb.com%2Fwp-content%2Fuploads%2FProcedure-Checklist-%25E2%2580%2593-Amplification-of-Full-Length-16S-Gene-with-Barcoded-Primers-for-Multiplexed-SMRTbell-Library-Preparation-and-Sequencing.pdf&data=05%7C02%7Cgerard.martinezdeleon%40unibe.ch%7C156efd6d37324a9244a208dc8f77c98d%7Cd400387a212f43eaac7f77aa12d7977e%7C1%7C0%7C638542991845812552%7CUnknown%7CTWFpbGZsb3d8eyJWIjoiMC4wLjAwMDAiLCJQIjoiV2luMzIiLCJBTiI6Ik1haWwiLCJXVCI6Mn0%3D%7C0%7C%7C%7C&sdata=udxelZhMLEBAReB2HrJQHxP72ahVm%2Bx2%2BmzaJvaTra8%3D&reserved=0) |
| Negative | MasterMix | [https://www.pacb.com/wp-content/uploads/Procedure-Checklist-%E2%80%93-Amplification-of-Full-Length-16S-Gene-with-Barcoded-Primers-for-Multiplexed-SMRTbell-Library-Preparation-and-Sequencing.pdf](https://eur03.safelinks.protection.outlook.com/?url=https%3A%2F%2Fwww.pacb.com%2Fwp-content%2Fuploads%2FProcedure-Checklist-%25E2%2580%2593-Amplification-of-Full-Length-16S-Gene-with-Barcoded-Primers-for-Multiplexed-SMRTbell-Library-Preparation-and-Sequencing.pdf&data=05%7C02%7Cgerard.martinezdeleon%40unibe.ch%7C156efd6d37324a9244a208dc8f77c98d%7Cd400387a212f43eaac7f77aa12d7977e%7C1%7C0%7C638542991845812552%7CUnknown%7CTWFpbGZsb3d8eyJWIjoiMC4wLjAwMDAiLCJQIjoiV2luMzIiLCJBTiI6Ik1haWwiLCJXVCI6Mn0%3D%7C0%7C%7C%7C&sdata=udxelZhMLEBAReB2HrJQHxP72ahVm%2Bx2%2BmzaJvaTra8%3D&reserved=0) |
| Positive | ATCC MSA-1010 | <https://www.atcc.org/products/msa-1010> |
| Positive | ZymoBIOMICS Microbial Community Standard | <https://zymoresearch.eu/products/zymobiomics-microbial-community-dna-standard-ii-log-distribution> |


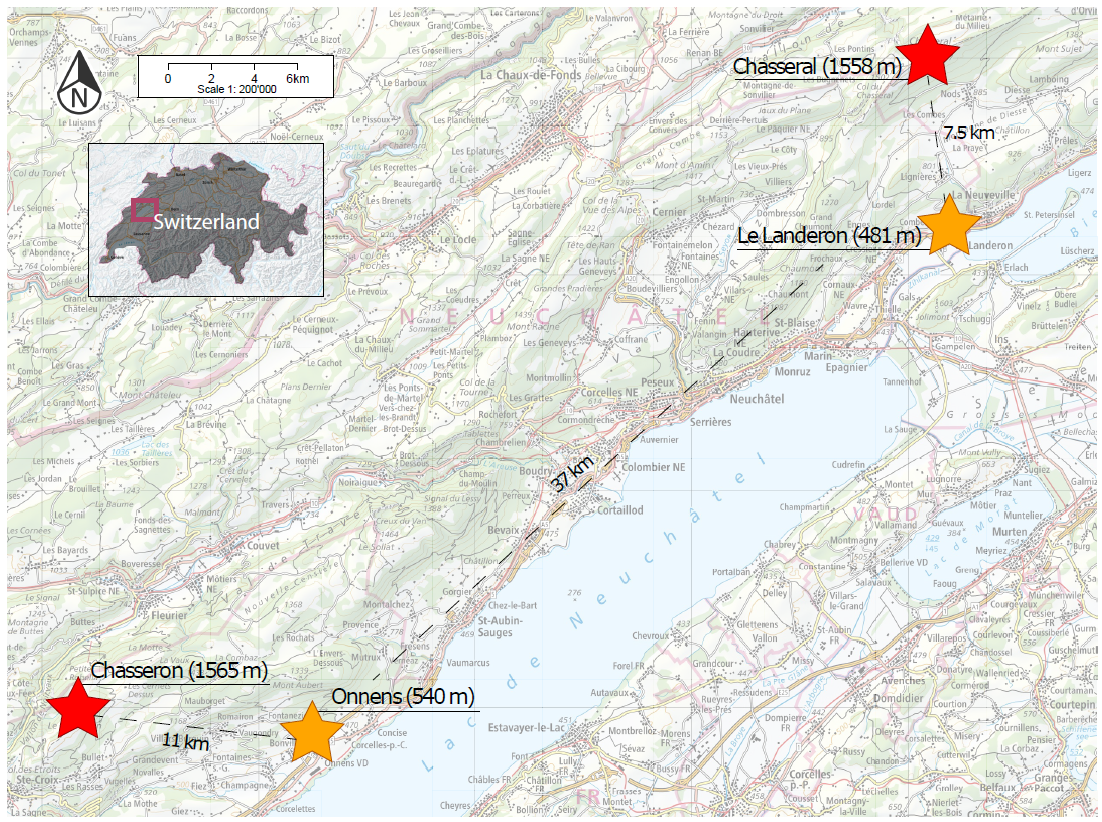


**Fig. S1.** Map of the study area showing the geographic position and elevation of the sampling sites, indicated with star signs. The shortest distance between sites of different blocks (north block: Chasseral and Le Landeron; south block: Chasseron and Onnens), as well as the distance between sites of the same block, are provided. Stars’ colors indicate sites at different elevations: red: high elevation; orange: low elevation. Map adapted from <https://map.geo.admin.ch>.


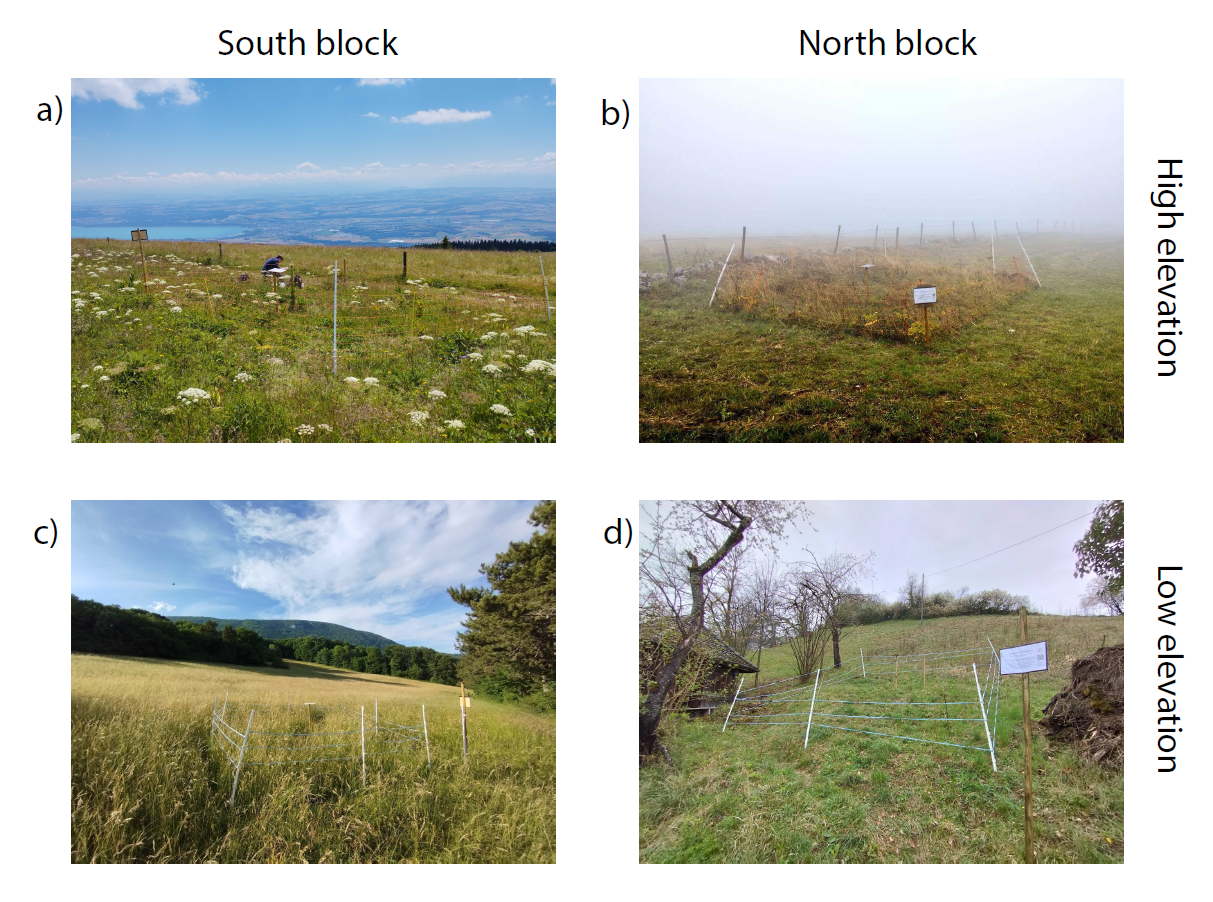


**Fig. S2.** Pictures of the field sites taken at various seasons: a) Chasseron (summer), b) Chasseral (autumn), c) Onnens (summer), d) Le Landeron (early spring, before the start of the experiments). The pictures are arranged in a grid, so that the rows indicate the elevation (high and low), and the columns show the block (south and north).


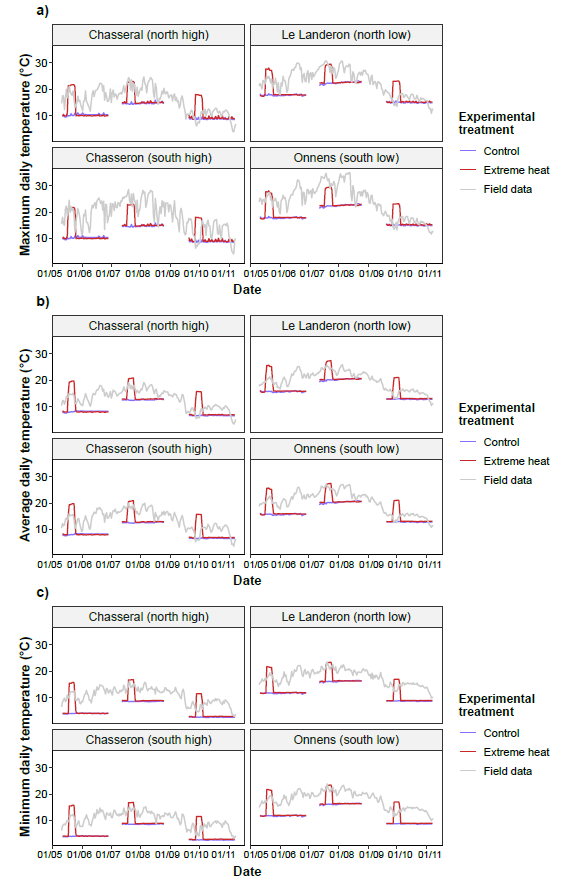


**Fig. S3.** Site-specific maximum (a), average (b) and minimum (c) daily soil temperatures at 5 cm depth, together with the daytime (a), average (b), and nighttime temperatures (c) recorded during the lab experiment, for both control (blue lines) and extreme heat treatments (red lines).


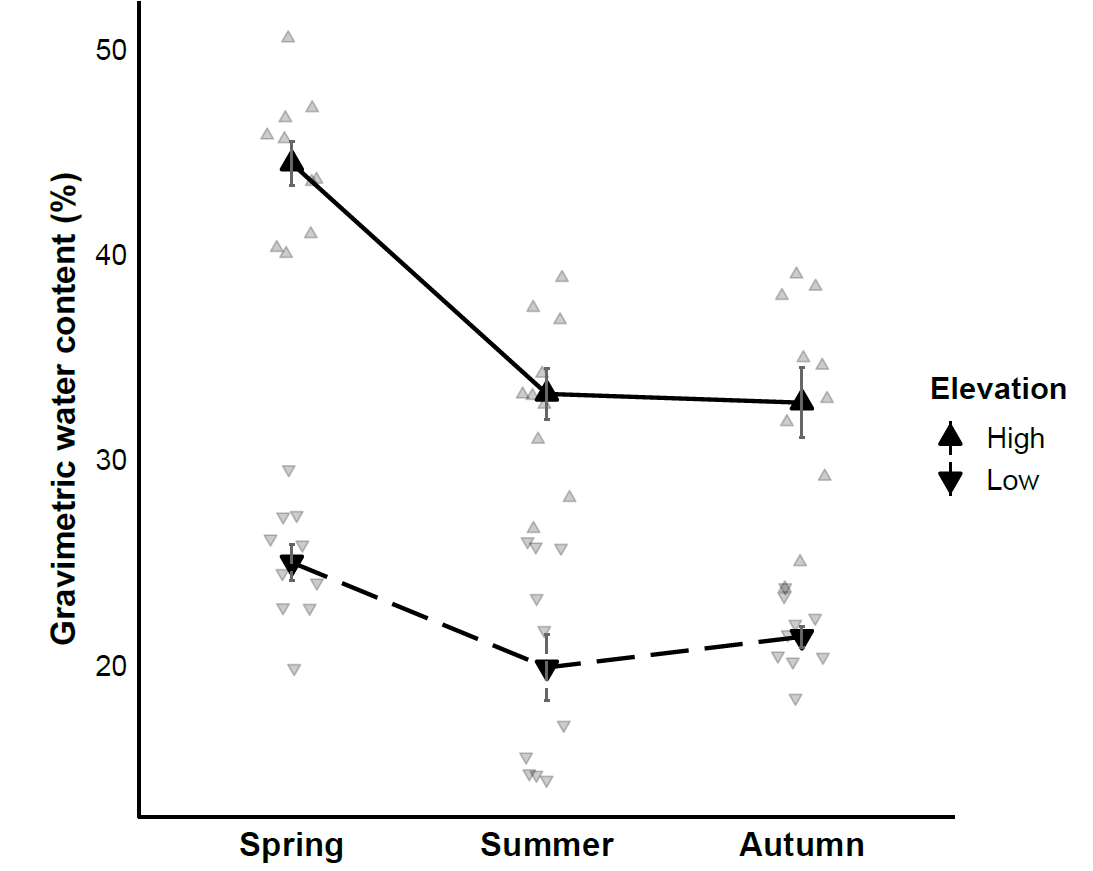


**Fig. S4.** Gravimetric soil water content, measured immediately after field sampling. Solid black points represent means, grey bars represent standard errors, and faded points are raw data (*N* = 10 per each elevation and season combination).


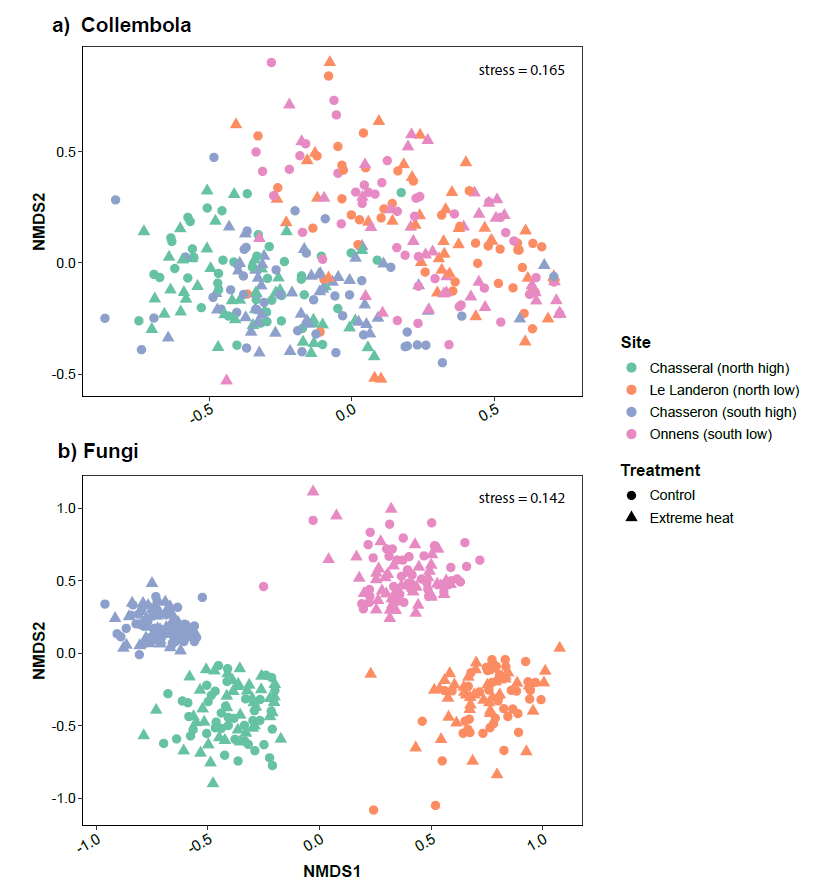


**Fig. S5.** Visualization of Collembola (a) and fungal communities (b) using non-metric multidimensional scaling (NDMS), implemented in the package *vegan* (version 2.6-4; Oksanen *et al.* 2022). Different colors indicate the sites: green: Chasseral; orange: Le Landeron; blue: Chasseron; pink: Onnens. The experimental treatments are shown with different shapes: round: control; triangle: extreme heat. We note that the first axis (NMDS1) mainly represents compositional differences between elevations (high/low), while the second axis (NMDS2) captures differences between the blocks (north/south). k=3 in both NDMS.

**
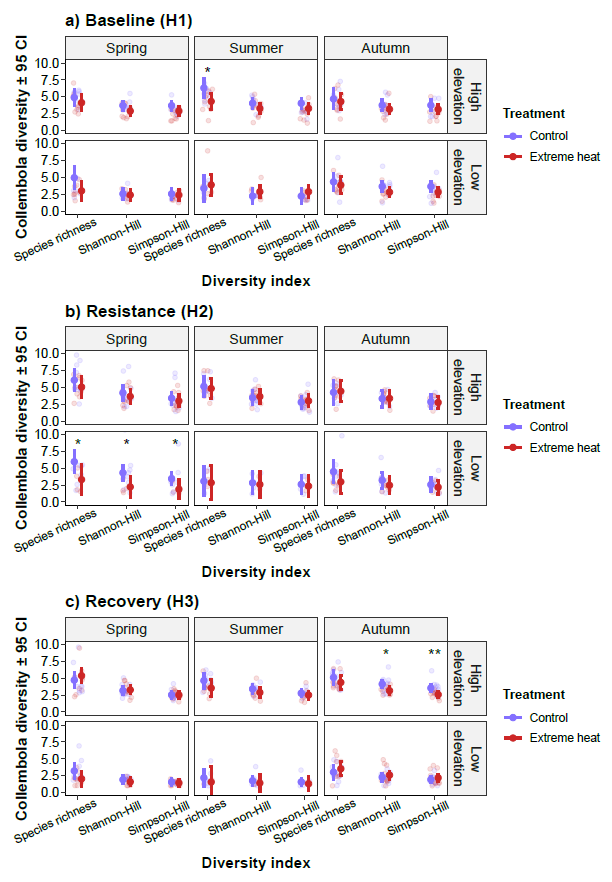
**

**Fig. S6.** Estimated marginal means (± 95 confidence intervals) of diversity profiles of Collembola communities, showing three indices calculated from various values of Hill number exponents (*q*): *q* = 0 (species richness), *q* = 1 (Shannon-Hill), *q* = 2 (Simpson-Hill). Lower values of the *q* exponent provide diversity estimates that give more leverage to rare species (e.g., species richness), while higher values give more leverage to dominant species (Roswell et al., 2021). Before calculating the diversity indices, we applied an abundance cut-off to restrict the diversity analysis to samples with at least ten individuals. Samples were equalized to a coverage value of 0.90. Diversity profiles are shown for each experimental harvest separately: a) baseline or harvest 1 (H1; *N* = 97), b) resistance or harvest 2 (H2; *N* = 91), and c) recovery or harvest 3 (H3; *N* = 103). Colours indicate different experimental temperature treatments: blue: control; red: extreme heat. Stars show significant differences between treatments at each harvest: **P* < 0.05, ***P* < 0.01.


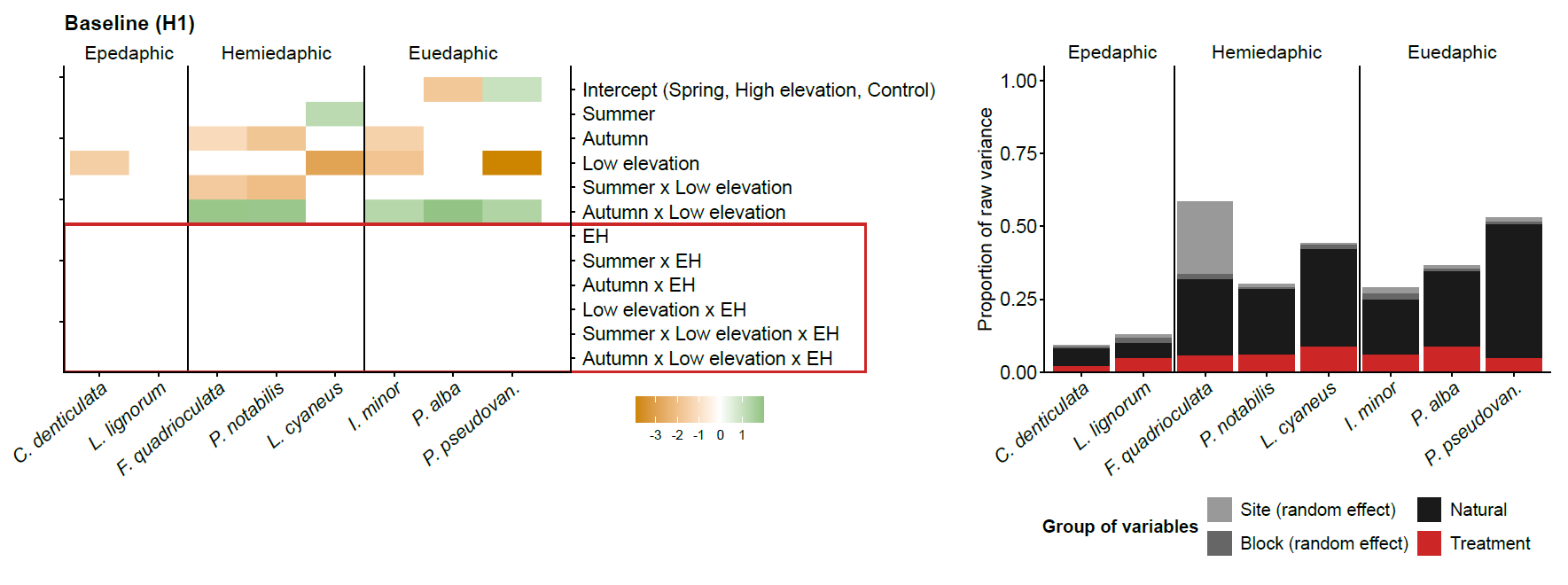


**Fig. S7.** Output of the joint species distribution models (jSDMs) fitted to investigate the responses of Collembola species abundances to season, elevation, treatment, and their three-way interactions, in the baseline response (i.e., harvest 1: H1; before the onset of the extreme heat events). Estimates from the beta parameters (left panels) show the responses of species abundances (x-axis) to each of the model parameters (y-axis). Green and orange colors indicate positive and negative responses with 95% posterior probability, respectively, while blank spaces denote responses that lacked statistical support. Species abundances at the intercept (spring, high elevation, control treatment) denote more abundant species in green, less abundant species in orange, and blank spaces indicating intermediate abundances. Parameters enclosed within the red area represent species responses to the experimental treatment (extreme heat: EH; see Table S6 for an ecological interpretation of the model parameters). The proportion of raw explained variance (right panels) is provided for different groups of variables: random effects (site and block), natural variables (season and elevation), and treatment (containing the variance explained by all parameters influenced by extreme heat, shown within the red area of the right panels). Collembola species are ordered according to their vertical stratification across the soil profile: epedaphic (surface-living), hemi-edaphic (living in litter and shallow soil layers), and euedaphic (permanently living in the soil).


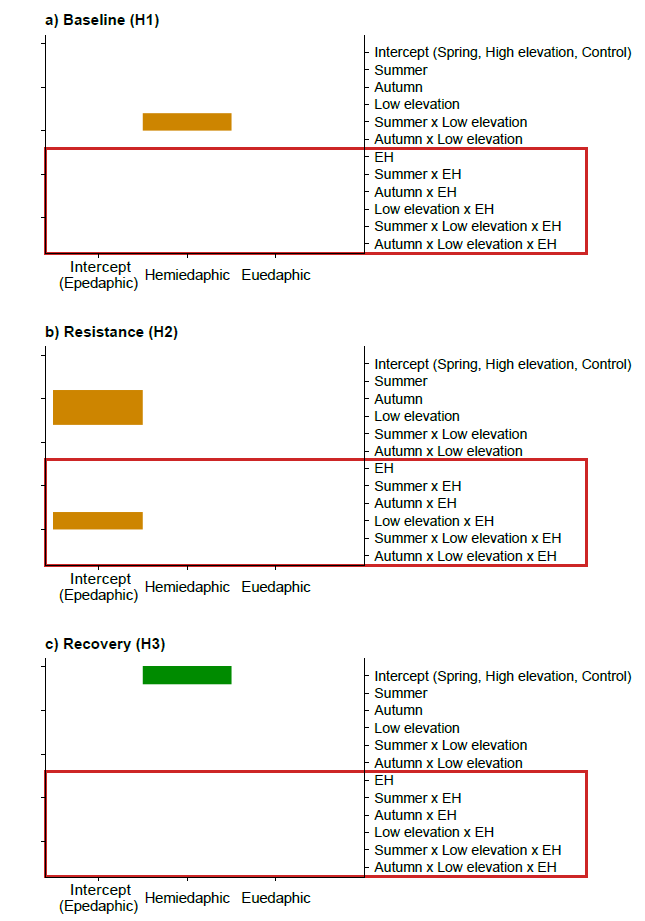


**Fig. S8.** Output of the joint species distribution models (jSDMs) fitted to investigate the responses of Collembola species abundances to season, elevation, treatment, and their three-way interactions, in the baseline (H1), resistance (H2) and recovery responses (H3). Estimates from the gamma parameters show whether species traits (i.e., vertical stratification; x-axis) mediate species abundance responses to each of the model parameters (y-axis). Three types of the vertical stratification of Collembola across the soil profile were investigated: epedaphic (surface-living), hemi-edaphic (living in litter and shallow soil layers), and euedaphic (permanently living in the soil). Green and orange colors indicate positive and negative responses with 95% posterior probability, respectively, while blank spaces denote responses that lacked statistical support. Parameter estimates at the intercepts (x-axis: epedaphic Collembola; y-axis: spring, high elevation, control treatment) denote higher overall abundances in green, lower overall abundances in orange, and blank spaces indicating intermediate abundances. The variation in species abundances explained by their vertical stratification ($R_{T}^{2}$; Ovaskainen *et al.* 2017) amounts to: 0.15 (baseline), 0.36 (resistance), 0.43 (recovery). Parameters enclosed within the red area represent species responses to the experimental treatment (extreme heat: EH; see Table S6 for an ecological interpretation of the model parameters).


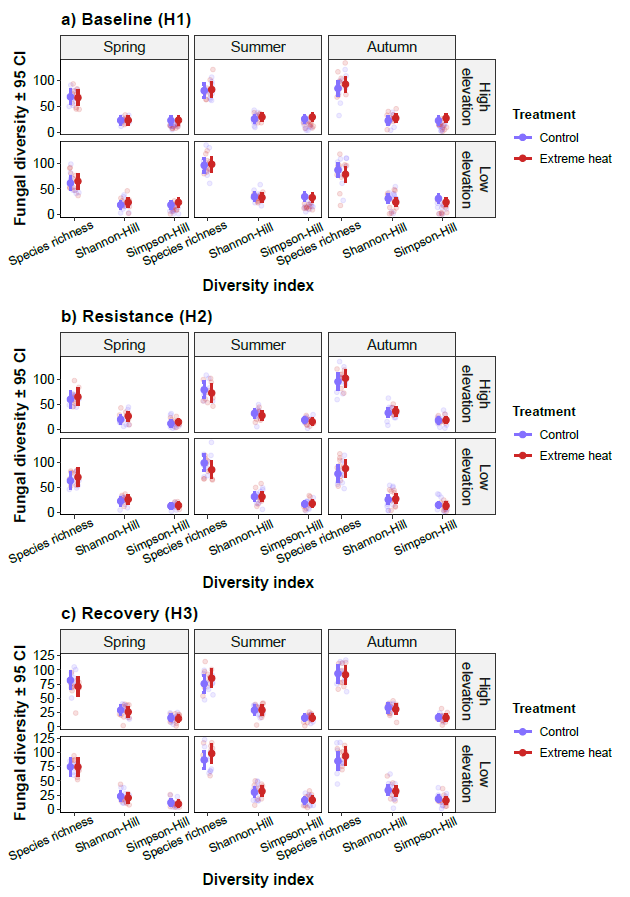


**Fig. S9.** Estimated marginal means (± 95 confidence intervals) of diversity profiles of fungal communities, showing three indices calculated from various values of Hill number exponents (*q*): *q* = 0 (species richness), *q* = 1 (Shannon-Hill), *q* = 2 (Simpson-Hill). Lower values of the *q* exponent provide diversity estimates that give more leverage to rare species (e.g., species richness), while higher values give more leverage to dominant species (Roswell et al., 2021). Samples were equalized to a coverage value of 0.98. Diversity profiles are shown for each experimental harvest separately: a) baseline or harvest 1 (H1; *N* = 120), b) resistance or harvest 2 (H2; *N* = 120), and c) recovery or harvest 3 (H3; *N* = 120). Colours indicate different experimental temperature treatments: blue: control; red: extreme heat. The extreme heat treatment did not have significant effects on fungal diversity in any case.


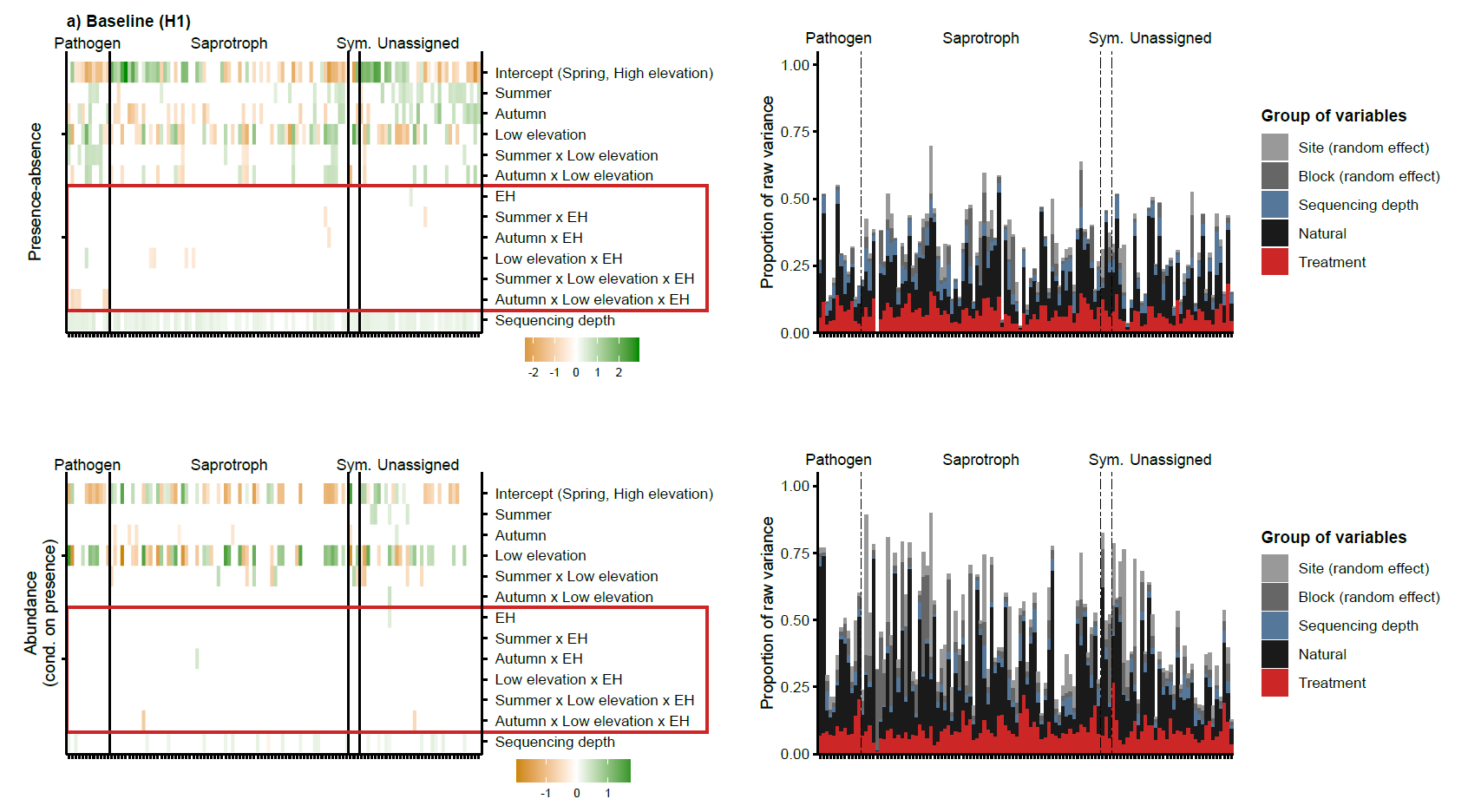


**Fig. S10.** Output of the joint species distribution models (jSDMs) fitted to investigate the responses of fungal species occurrences (panels above) and relative abundances (panels below) to season, elevation, treatment, and their three-way interactions, in the baseline response (i.e., harvest 1: H1; before the onset of the extreme heat events). Estimates from the beta parameters (left panels) show the species responses (x-axis) to each of the model parameters (y-axis). Green and orange colors indicate positive and negative responses with 95% posterior probability, respectively, while blank spaces denote responses that lacked statistical support. Species abundances at the intercept (spring, high elevation, control treatment) denote more abundant species in green, less abundant species in orange, and blank spaces indicating intermediate abundances. Parameters enclosed within the red area represent species responses to the experimental treatment (extreme heat: EH; see Table S6 for an ecological interpretation of the model parameters). The proportion of raw explained variance (right panels) is provided for different groups of variables: random effects (site and block), natural variables (season and elevation), and treatment (containing the variance explained by all parameters influenced by extreme heat, shown within the red area of the right panels). Fungal species are ordered according to their main trophic modes: pathogens, saprotrophs, symbionts, and unassigned fungi.


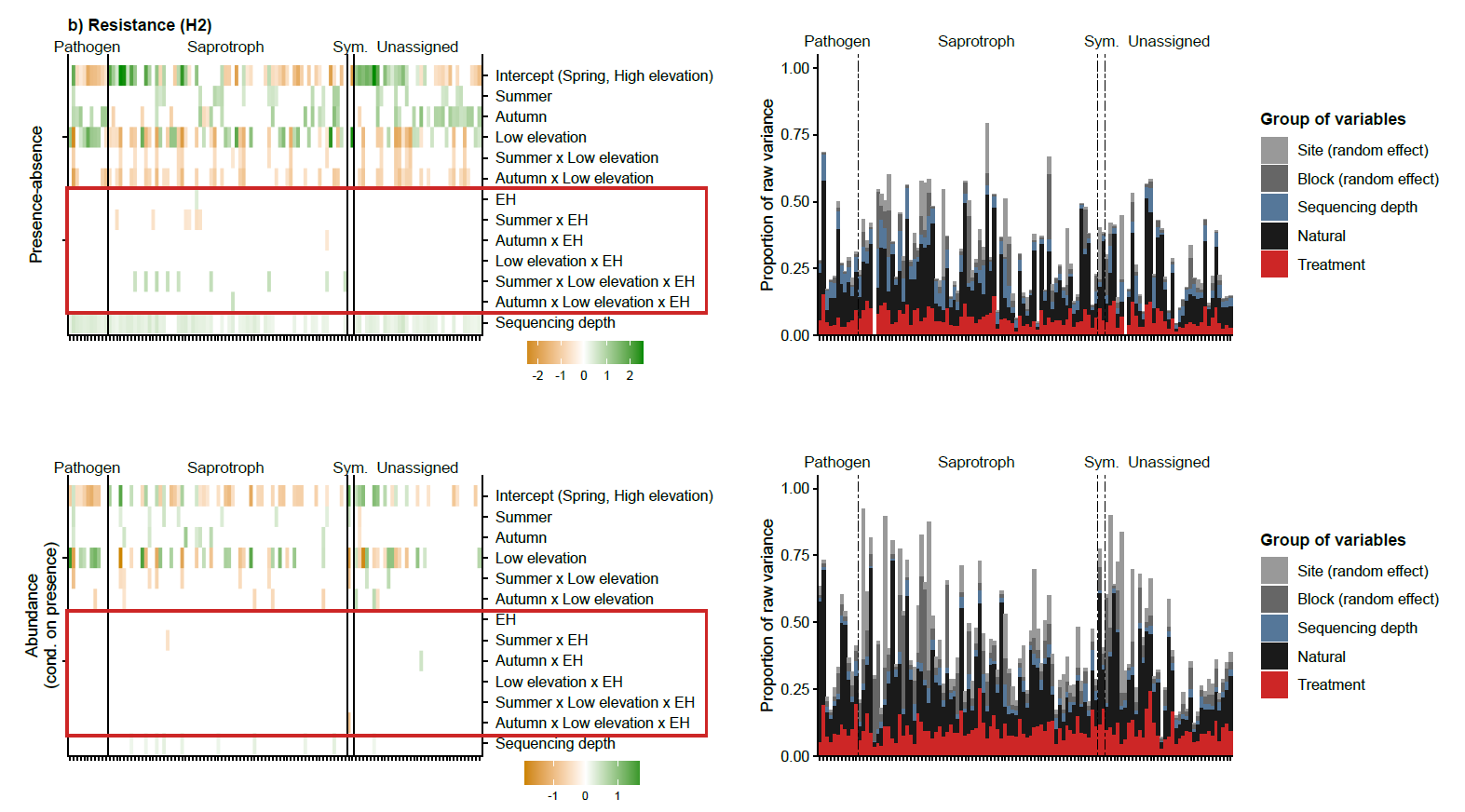


**Fig. S11.** Output of the joint species distribution models (jSDMs) fitted to investigate the responses of fungal species occurrences (panels above) and relative abundances (panels below) to season, elevation, treatment, and their three-way interactions, in the resistance response (i.e., harvest 2: H2; after the extreme heat events). Estimates from the beta parameters (left panels) show the species responses (x-axis) to each of the model parameters (y-axis). Green and orange colors indicate positive and negative responses with 95% posterior probability, respectively, while blank spaces denote responses that lacked statistical support. Species abundances at the intercept (spring, high elevation, control treatment) denote more abundant species in green, less abundant species in orange, and blank spaces indicating intermediate abundances. Parameters enclosed within the red area represent species responses to the experimental treatment (extreme heat: EH; see Table S6 for an ecological interpretation of the model parameters). The proportion of raw explained variance (right panels) is provided for different groups of variables: random effects (site and block), natural variables (season and elevation), and treatment (containing the variance explained by all parameters influenced by extreme heat, shown within the red area of the right panels). Fungal species are ordered according to their main trophic modes: pathogens, saprotrophs, symbionts, and unassigned fungi.


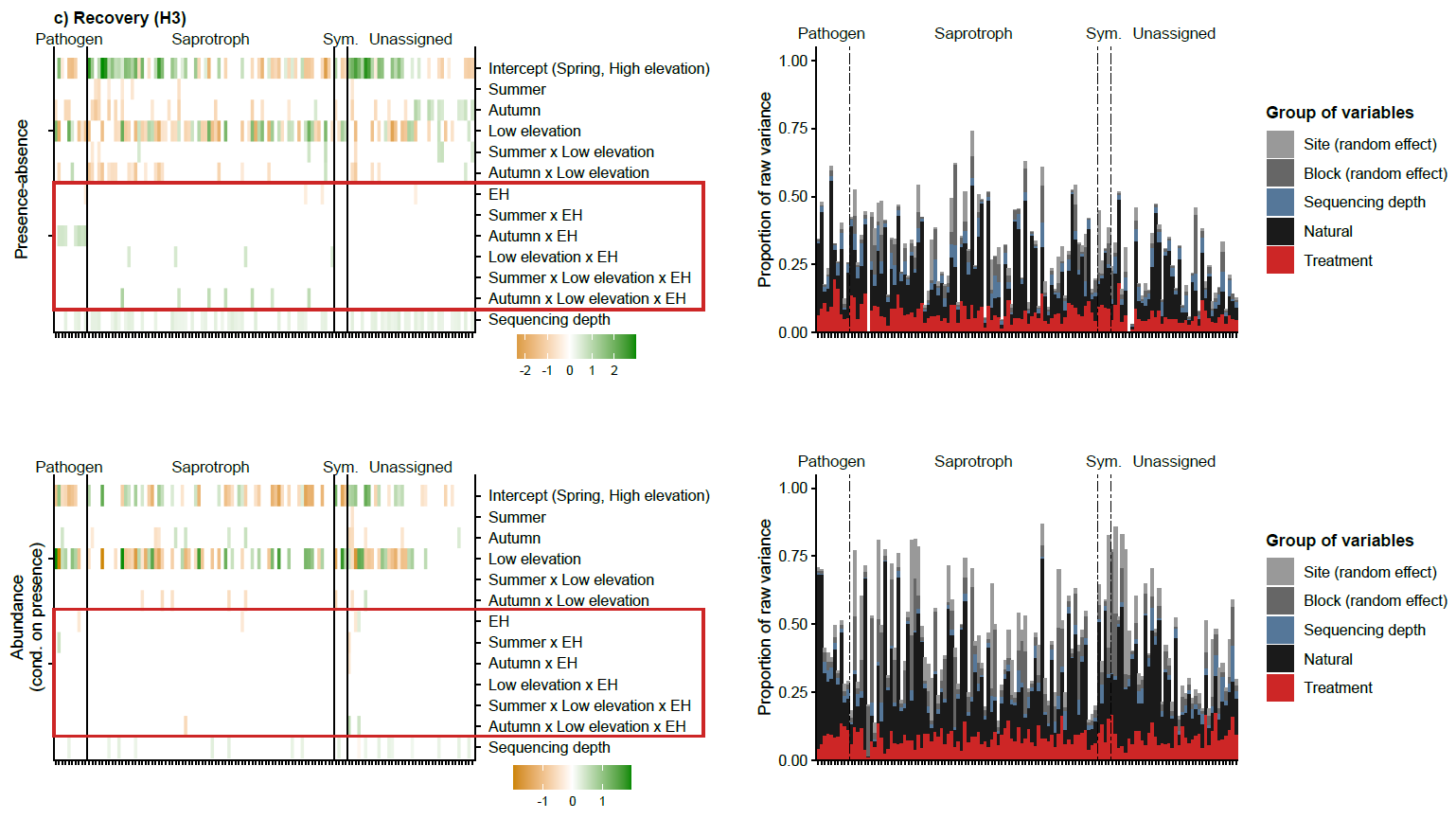


**Fig. S12.** Output of the joint species distribution models (jSDMs) fitted to investigate the responses of fungal species occurrences (panels above) and relative abundances (panels below) to season, elevation, treatment, and their three-way interactions, in the recovery response (i.e., harvest 3: H3; five weeks after the end of the extreme heat events). Estimates from the beta parameters (left panels) show the species responses (x-axis) to each of the model parameters (y-axis). Green and orange colors indicate positive and negative responses with 95% posterior probability, respectively, while blank spaces denote responses that lacked statistical support. Species abundances at the intercept (spring, high elevation, control treatment) denote more abundant species in green, less abundant species in orange, and blank spaces indicating intermediate abundances. Parameters enclosed within the red area represent species responses to the experimental treatment (extreme heat: EH; see Table S6 for an ecological interpretation of the model parameters). The proportion of raw explained variance (right panels) is provided for different groups of variables: random effects (site and block), natural variables (season and elevation), and treatment (containing the variance explained by all parameters influenced by extreme heat, shown within the red area of the right panels). Fungal species are ordered according to their main trophic modes: pathogens, saprotrophs, symbionts, and unassigned fungi.

**
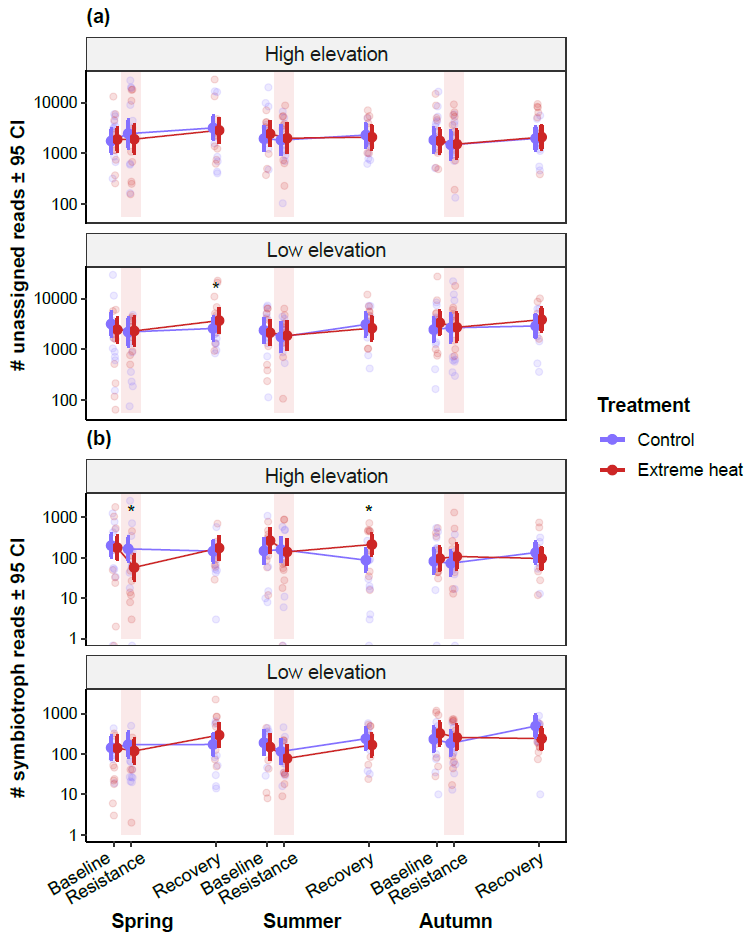
**

**Fig. S13.** Estimated marginal means (± 95 confidence intervals) of the number of reads (log-transformed) of unassigned (a; upper panel) and symbiotic fungi (b; lower panel) over the course of the experiments in spring, summer and autumn. We note that the number of reads represent relative abundances given the compositionality of metabarcoding data. The labels on the x-axis specify the different time points in which fungal metabarcoding reads were assessed during the experiment (i.e., harvests): baseline (harvest 1); resistance (harvest 2); recovery (harvest 3). The faded red areas represent the one-week extreme heat events. Colours indicate different experimental temperature treatments: blue: control; red: extreme heat. Stars show significant differences between treatments at each harvest: **P* < 0.05.


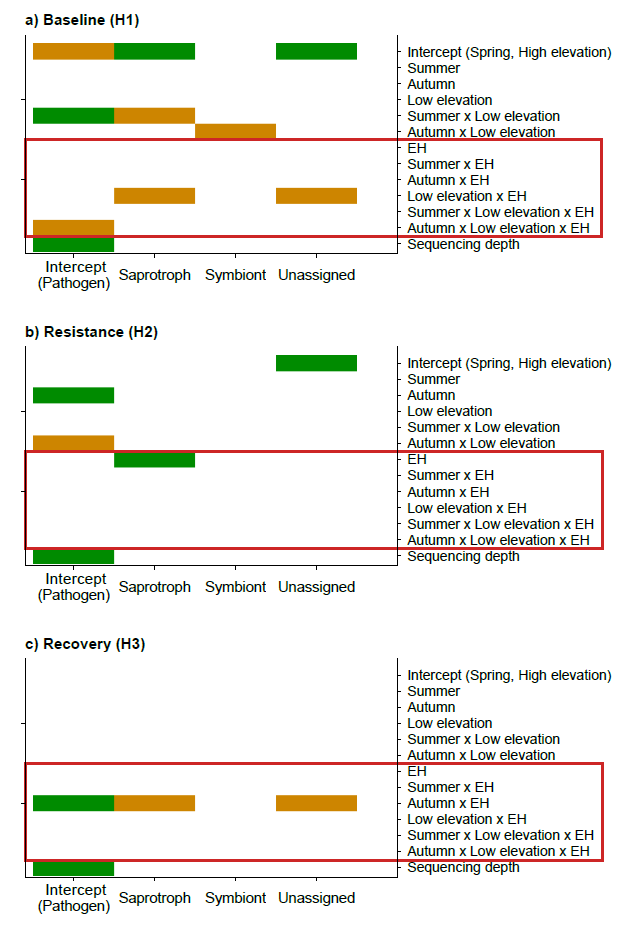


**Fig. S14.** Output of the joint species distribution models (jSDMs) fitted to investigate the responses of fungal species occurrences (i.e., presence-absence) to season, elevation, treatment, and their three-way interactions, in the baseline (H1), resistance (H2) and recovery responses (H3). Estimates from the gamma parameters show whether species traits (i.e., fungal trophic modes; x-axis) mediate species occurrence responses to each of the model parameters (y-axis). Three types of the fungal trophic modes were investigated: pathogens, saprotrophs and symbionts. Unassigned fungi represent the species for which a trophic mode could not be reliably determined. Green and orange colors indicate positive and negative responses with 95% posterior probability, respectively, while blank spaces denote responses that lacked statistical support. Parameter estimates at the intercepts (x-axis: pathogenic fungi; y-axis: spring, high elevation, control treatment) denote higher overall occurrences in green, lower overall occurrences in orange, and blank spaces indicating intermediate occurrences. The variation in species occurrences explained by their trophic modes ($R_{T}^{2}$; Ovaskainen *et al.* 2017) amounts to: 0.08 (baseline), 0.06 (resistance), 0.06 (recovery). Parameters enclosed within the red area represent species responses to the experimental treatment (extreme heat: EH; see Table S6 for an ecological interpretation of the model parameters).


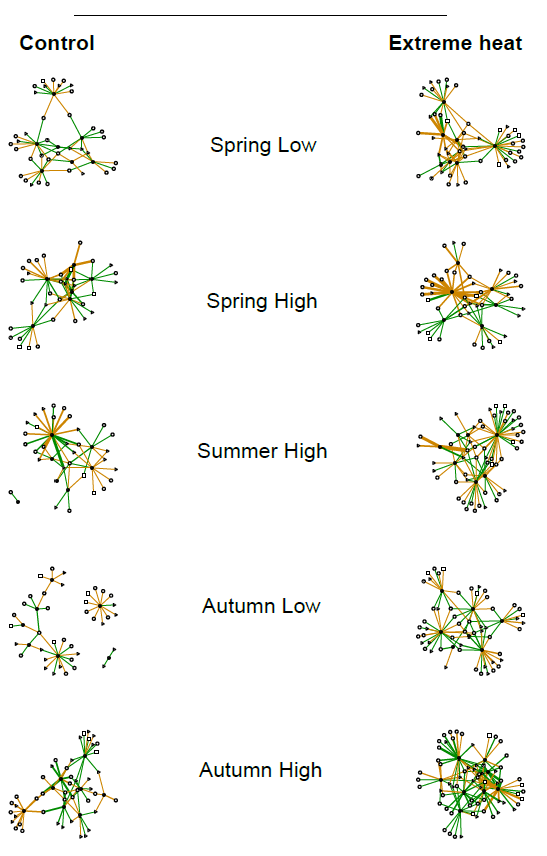


**Fig. S15.** Association networks of Collembola and fungi in the different spatiotemporal contexts (i.e., season and elevation) at the recovery response, separately for control (left column) and extreme heat treatments (right column). Positive associations are displayed with green colors and negative associations are shown with orange colors. The width of the links is proportional to the strength of the associations (i.e., parameter estimates of the Collembola-fungal jSDM). Black and white nodes denote Collembola and fungal species, respectively. Different node shapes represent various fungal trophic groups: saprotrophs (circle), pathogens (square), symbionts (pie), and unassigned fungi (triangle). Nodes without associations (i.e., degree = 0) are not displayed. A high-resolution version of the figure is available at the data repository (*Data from: Belowground Communities in Lowlands Are Less Stable to Climate Extremes across Seasons*, 2025).

**SI References**

Blanchet, F. G., Cazelles, K., & Gravel, D. (2020). Co-occurrence is not evidence of ecological interactions. *Ecology Letters*, *23*(7), 1050–1063. https://doi.org/10.1111/ele.13525

Chauvat, M., Perez, G., & Ponge, J. F. (2014). Foraging patterns of soil springtails are impacted by food resources. *Applied Soil Ecology*, *82*, 72–77. https://doi.org/10.1016/j.apsoil.2014.05.012

Csárdi, G., Nepusz, T., Traag, V., Horvát, S., Zanini, F., Noom, D., & Müller, K. (2024). *igraph: Network Analysis and Visualization in R* [Computer software]. https://doi.org/10.5281/zenodo.7682609

*Data from: Belowground communities in lowlands are less stable to climate extremes across seasons*. (2025). [Dataset]. Figshare Repository. https://doi.org/10.6084/m9.figshare.26142490

Dormann, C. F., Frund, J., Bluthgen, N., & Gruber, B. (2009). Indices, Graphs and Null Models: Analyzing Bipartite Ecological Networks. *The Open Ecology Journal*, *2*(1), 7–24. https://doi.org/10.2174/1874213000902010007

Dormann, C. F., Gruber, B., & Fründ, J. (2008). *Introducing the bipartite Package: Analysing Ecological Networks*. *8/2*, 8–11.

Dunger, W., & Schlitt, B. (2011). Synopses on Palaearctic Collembola – Tullbergiidae. *Soil Organisms*, *83*(1), Article 1.

Erktan, A., Or, D., & Scheu, S. (2020). The physical structure of soil: Determinant and consequence of trophic interactions. *Soil Biology and Biochemistry*, *148*, 107876. https://doi.org/10.1016/j.soilbio.2020.107876

Ferlian, O., Klarner, B., Langeneckert, A. E., & Scheu, S. (2015). Trophic niche differentiation and utilisation of food resources in collembolans based on complementary analyses of fatty acids and stable isotopes. *Soil Biology and Biochemistry*, *82*, 28–35. https://doi.org/10.1016/j.soilbio.2014.12.012

Fjellberg, A. (1998). *The Collembola of Fennoscandia and Denmark, Part I: Poduromorpha* (Vol. 35, p. 184). Brill. https://brill.com/display/title/6555

Fjellberg, A. (2007). *The Collembola of Fennoscandia and Denmark, Part II: Entomobryomorpha and Symphypleona* (Vol. 42). Brill. https://brill.com/display/title/14147

Gisin, H. (1960). *Collembolenfauna Europas*. Museum D’Histoire Naturelle Genève.

Gisin, H. F. (1943). *Oekologie und Lebensgemeinschaften der Collembolen im schweizerischen Exkursionsgebiet Basels*. Kundig. https://books.google.ch/books?id=m5HFzQEACAAJ

Hopkin, S. P. (2007). *A Key to the Collembola (springtails) of Britain and Ireland*. FSC.

Jackson, M. C., Pawar, S., & Woodward, G. (2021). The Temporal Dynamics of Multiple Stressor Effects: From Individuals to Ecosystems. *Trends in Ecology and Evolution*, *36*(5), 402–410. https://doi.org/10.1016/j.tree.2021.01.005

Leinaas, H. P., & Bleken, E. (1983). Egg diapause and demographic strategy in *Lepidocyrtus lignorum* Fabricius (Collembola; Entomobryidae). *Oecologia*, *58*(2), 194–199.

Makowski, D., Ben-Shachar, M., & Lüdecke, D. (2019). bayestestR: Describing Effects and their Uncertainty, Existence and Significance within the Bayesian Framework. *Journal of Open Source Software*, *4*(40), 1541. https://doi.org/10.21105/joss.01541

Martínez-De León, G., & Thakur, M. P. (2024). Ecological debts induced by heat extremes. *Trends in Ecology and Evolution*, *39*(11), 1024–1034. https://doi.org/10.1016/j.tree.2024.07.002

May, R. M. (1972). Will a Large Complex System be Stable? *Nature*, *238*(8), 413–414. https://doi.org/10.1111/ele.14242

Nakagawa, S., & Schielzeth, H. (2013). A general and simple method for obtaining R2 from generalized linear mixed-effects models. *Methods in Ecology and Evolution*, *4*(2), 133–142. https://doi.org/10.1111/j.2041-210x.2012.00261.x

Oksanen, J., Simpson, G., Blanchet, F., Kindt, R., Legendre, P., Minchin, P., O’Hara, R., Solymos, P., Stevens, M., Szoecs, E., Wagner, H., Barbour, M., Bedward, M., Bolker, B., Borcard, D., Carvalho, G., Chirico, M., De Caceres, M., Durand, S., … Weedon, J. (2022). *vegan: Community Ecology Package* [Computer software]. https://cran.r-project.org/package=vegan

Ovaskainen, O., Tikhonov, G., Norberg, A., Guillaume Blanchet, F., Duan, L., Dunson, D., Roslin, T., & Abrego, N. (2017). How to make more out of community data? A conceptual framework and its implementation as models and software. *Ecology Letters*, *20*(5), 561–576. https://doi.org/10.1111/ele.12757

Pinheiro, J., Bates, B., & R Core Team. (2023). *nlme: Linear and Nonlinear Mixed Effects Models* [Computer software]. https://cran.r-project.org/package=nlme

Poisot, T., Canard, E., Mouillot, D., Mouquet, N., & Gravel, D. (2012). The dissimilarity of species interaction networks. *Ecology Letters*, *15*(12), 1353–1361. https://doi.org/10.1111/ele.12002

Roswell, M., Dushoff, J., & Winfree, R. (2021). A conceptual guide to measuring species diversity. *Oikos*, *130*(3), 321–338. https://doi.org/10.1111/oik.07202

Thibaud, J.-M., Schulz, H.-J., & da Gama, M. M. (2004). *Synopses on Palaearctic Collembola, Volume IV: Hypogastruridae* (Vol. 75). https://www.nhbs.com/en/synopses-on-palaearctic-collembola-volume-4-hypogastruridae-book

Urbášek, F., & Rusek, J. (1994). Activity of digestive enzymes in seven species of Collembola (Insecta: Entognatha). *Pedobiologia*, *38*(5), 400–406. https://doi.org/10.1016/s0031-4056(24)00143-4
